# Cross-reactive antibody and T-cell responses after influenza virus infection in community-dwelling older adults

**DOI:** 10.1128/jvi.00407-26

**Published:** 2026-05-12

**Authors:** Lisa van Pul, Sietske Karla Rosendahl Huber, Ronald Jacobi, Marion Hendriks, Roos van Schuijlenburg, Yannick van Sleen, Elske Bijvank, Jelle de Wit, Josine van Beek

**Affiliations:** 1 Centre for Infectious Disease Control, National Institute for Public Health and the Environment (RIVM) 10206, Bilthoven, the Netherlands; Fred Hutchinson Cancer Center Vaccine and Infectious Disease Division, Seattle, Washington, USA

**Keywords:** influenza virus, older adults, humoral response, cellular response, cross-reactivity

## Abstract

Seasonal influenza virus infection is one of the main causes of morbidity and mortality in older adults. The high mutation rate of influenza virus gives rise to new variants that are antigenically different, thereby evading pre-existing immune responses. In addition, due to aging of the immune system, older adults have diminished responses to infections with novel antigens. Here, we investigated longitudinal influenza virus-specific humoral and cellular responses in a cohort of community-dwelling older adults after influenza virus infection in the 2014/15 flu season in the Netherlands, a season hallmarked by vaccine mismatch. Moreover, we assessed the effect of vaccination on these responses and potential cross-reactivity. Hemagglutination inhibition assays revealed that antibodies against both vaccine and circulating strains increased 2 weeks after infection, regardless of vaccination status, indicating cross-reactivity. Although infection alone was capable of inducing antibodies, prior vaccination further increased titers. Peripheral blood mononuclear cell (PBMC) stimulation with hemagglutinin-peptide pools of circulating and vaccine strains showed induction of strain-specific T-cell responses after infection in IFN-γ ELISpots. At each timepoint, T-cell responses were similar between individuals vaccinated prior to infection and unvaccinated individuals. However, the increase in influenza virus-specific T-cell responses from 72 h to 2 weeks post-infection seemed stronger in individuals vaccinated before infection. Moreover, despite mismatch between vaccine and circulating strains, T-cell cross-reactivity was observed, albeit less pronounced than the antibody response. Overall, our study shows that community-dwelling older adults with influenza virus infection are capable of inducing virus-specific immune responses with cross-reactive potential, and vaccination further enhances these responses.

**IMPORTANCE:**

The older population is at increased risk for severe influenza virus infection due to age-related impaired immune responses to novel antigens. Consequently, older adults rely more on previously formed cross-reactive memory responses compared to younger adults. In this study, we longitudinally investigated the influenza virus-specific antibody response as well as the virus-specific T-cell response in community-dwelling older adults after influenza virus infection. We demonstrate that, despite higher age, this group of older adults was capable of inducing influenza virus-specific antibody and T-cell responses. Moreover, receiving vaccination prior to infection further increased these immune responses. Importantly, individuals mounted antibody and T-cell responses to several different influenza virus strains, indicating cross-reactivity at both levels, despite mismatch between vaccine and infection strains. Unraveling the immune responses to influenza virus infection in older adults could inform new vaccine developments and strategies that may better protect the vulnerable older population from infection.

## INTRODUCTION

The immune system is under repeated pressure from seasonal recirculation of influenza viruses. Every year, around 3–5 million people experience a severe influenza virus infection, and approximately 290,000 to 650,000 respiratory deaths are caused by influenza virus infection worldwide (1, 2). Furthermore, it is one of the major causes of morbidity and mortality among older adults (3). Seasonal recirculation of influenza virus is due to antigenic drift, where point mutations are introduced in the viral genome, particularly in the two main surface antigens of influenza virus, hemagglutinin (HA) and neuraminidase (NA) (4, 5). This can lead to the emergence of novel, antigenically different subclades that can evade previously built-up host immunity and cause seasonal influenza virus re-infections (6–8).

An efficient anti-viral immune response against influenza virus is highly reliant on adaptive immune responses. B-cells play an important role by producing neutralizing antibodies targeting influenza virus antigens. Antibodies recognizing epitopes in HA and NA can protect against infection and have previously been associated with decreased disease severity (9–11). The T-cell response also plays a critical role in controlling and clearing influenza virus infection and has been shown to be a better correlate of protection against influenza virus infection in older adults than antibody titers (12, 13). CD8 T-cells can recognize infected cells and produce cytotoxic cytokines, which then eliminate these cells. In addition, CD4 T-cells can further expand the CD8 T-cell response and assist in virus-specific CD8 memory T-cell generation (14, 15). CD4 T-cells are also capable of producing cytotoxic cytokines themselves, as well as activate B cells and support the production of antibodies (14–16). Furthermore, where antibodies mainly target the highly variable head of HA on the surface of the virus, T-cells can mount responses to the more conserved internal viral proteins, such as matrix 1 protein (M1) (17–19). New viral variants that arise due to mutation of influenza virus can evade antibody binding; however, pre-existing T-cell responses may still cross-react to different epitopes on the virus that have been preserved (20–23).

However, with age, the immune system becomes gradually more dysfunctional, in a process called immunosenescence. Hallmarks of immunosenescence include a chronic inflammatory state, as well as a reduced output of naïve B and T lymphocytes due to diminished bone marrow output and thymic involution, respectively, resulting in a shift toward more memory lymphocytes specific for pathogens encountered earlier in life (24–26). As a result, there is a narrowing of the immune repertoire with loss of B- and T-cell diversity, as well as the impaired ability to produce high-affinity neutralizing antibody responses (25, 26). Immunosenescence also results in a loss of T-cell functionality, with reduced proliferative capacity and diminished cytokine production, which compromises clearance of viruses (3, 27). Indeed, this has also been shown in the influenza virus-specific T-cell response, where a reduction in T-cell function was observed in older adults, and this was associated with age-related lower T-cell receptor diversity (TCR) (28, 29). Furthermore, mutated influenza virus peptides were recognized less well by the TCRs of older adults (28). The impaired ability to induce a *de novo* immune response, for example to a new variant of influenza virus, could thus lead to an increased susceptibility, resulting in higher morbidity and mortality from influenza virus infection in this age group (3, 30, 31). As a consequence, older adults are more heavily dependent on cross-reactive memory B- and T-cell responses that were generated earlier in life to protect against new antigenically drifted strains.

Currently, vaccination is the best strategy to prevent influenza virus infection, as well as severe disease and death as a result of this infection (1). In the Netherlands, seasonal influenza vaccination is offered to all adults aged 60 years and older and to people at high risk of complications from influenza virus infection. The majority of the seasonal influenza virus infections are either caused by influenza virus type A or B. Type A viruses can be classified into subtypes based on the HA and NA surface proteins and include strains such as the currently circulating strains A(H1N1) and A(H3N2), which can be further divided into subclades (32). Type B viruses are divided into lineages based only on HA, such as B/Yamagata lineage and B/Victoria lineage. The seasonal influenza vaccine usually includes an influenza A(H1N1) virus strain, an influenza A(H3N2), and one or two influenza B virus strains from the B/Yamagata or B/Victoria lineage. Although vaccines against influenza are updated routinely, strains in the vaccine do not always perfectly match the circulating strains. In this study, we describe the influenza virus-specific immune responses in older adults in samples collected during the influenza season of 2014/15 in the Netherlands, a season hallmarked by vaccine mismatch. Whether vaccine and circulating strains are antigenically related is typically determined by antibody-mediated hemagglutination inhibition (HI) assay (33). In the influenza season of 2014/15, the circulating influenza A(H1N1) strain and the A(H1N1) strain in the vaccine were antigenically similar (32). However, in this season, there was a mismatch between the A(H3N2) vaccine strain and circulating A(H3N2) strains in the Netherlands due to antigenic drift. The main circulating A(H3N2) strains in that season in the Netherlands were from the 3C.3b and 3C.2a subclades, whereas the vaccine strain was from subclade 3C.1 (32, 34). Although the HA protein from the 3C.3b subclade did not antigenically differ from the A(H3N2) vaccine strain in HI assays (32, 33, 35), the HA protein from the 3C.2a subclade did (32, 35). In addition, the selected influenza B virus in the vaccine was also not optimally comparable to the circulating variant (32). Consequently, the vaccine mismatch contributed to low vaccine effectiveness and a relatively severe season with high excess mortality (32).

The mismatch between circulating influenza virus strains and the vaccine strain provided a unique opportunity to further investigate the longitudinal humoral and cellular cross-reactivity in our Dutch influenza-infection cohort of community-dwelling older adults from the 2014/15 influenza season. The antibody and T-cell responses to vaccine and circulating strains were investigated as well as their cross-reactive capacity. Furthermore, the effect of vaccination on these responses was assessed as well as the relation of the immune responses to symptom burden. An improved understanding of how the immune system of older adults responds to infection and vaccination can contribute to guiding new efforts to protect the aging population from infectious diseases.

## RESULTS

### Characteristics of the study cohort

In the current analysis, community-dwelling older individuals with laboratory-confirmed and symptomatic influenza virus infection were included from the original ILI-3 cohort, which was conducted during the 2014/15 influenza season. The results and design of the whole ILI-3 study have previously been described (34, 36). Briefly, in the ILI-3 cohort, older individuals were asked to monitor and report influenza-like illness (ILI) events during the influenza season. In case of an ILI event, as defined by the Dutch Pel criteria (37), participants were sampled within 72 h after symptom onset as well as after 2 and 8 weeks of ILI onset. Out of all the ILI-3 participants, 100 had a laboratory-confirmed influenza virus infection as determined by analysis of the RNA isolated from nasopharyngeal and oropharyngeal swabs. Based on the characteristics described in Tables 1 and 2, the 100 individuals are representative of the whole ILI-3 cohort (34). As reported previously, subtyping of influenza virus in this group revealed that 76% of individuals were infected with influenza A, the majority of which consisted of A(H3N2) infections (89.5%), and 10.5% were of the A(H1N1) strain (34). Out of the A(H3N2) infections, 39 individuals (57.3%) were infected with HA genetic clade 3C.2a, 22 (32.3%) were infected with clade 3C.3b, and for 7 individuals (10.3%) the subclade could not further be determined (Table 1; previously published in [34]). Finally, there were 24 influenza B virus (IBV) infections, all of the B/Yamagata lineage. When comparing the different influenza virus infection strains, no significant differences were found in age or sex of the participants (34). In addition, characteristics were compared between individuals vaccinated in the 2014/15 season prior to infection and individuals that were not vaccinated in the 2014/15 season. Of the 100 individuals, 69 received the seasonal influenza vaccination of 2014/15 and 31 did not (Table 2). The proportion of male and female participants did not significantly differ between the two groups. However, in line with previous publication (34), median age was significantly higher in vaccinated individuals as compared to unvaccinated individuals (Table 2). All vaccinated individuals included in the study had been vaccinated at least once before receiving vaccination in the 2014/15 season and >80% had received a vaccination in all of the previous 5 years (Table S1). Out of the 31 unvaccinated individuals, only five have received a vaccination in the five influenza seasons prior to the one described in this study; however, none were vaccinated in the directly preceding season (2013/14). Furthermore, a limited number of viral co-infections were noted in our participant group (Table S2).

**TABLE 1:** Baseline characteristics of the study population^*c*^

	*N*	Median age in years (IQR)	Female, *n* (%)
A(H1N1)	8	66 (64.3–69.5)	4 (50%)
A(H3N2)	68	70 (66–74)	29 (42.6%)
3C.2a	39	70 (66–74)	18 (46.2%)
3C.3b	22	67 (64–73.3)	9 (40.9%)
Not typable	7	71 (66–78)	2 (28.6%)
B/Yamagata	24	68 (64.3–73)	10 (41.7%)
*P*-value		0.320^*a*^	0.914^*b*^

^*a*^ Kruskal-Wallis.

^*b*^ Pearson χ.

^*c*^ Kaaijk et al. (34).

**TABLE 2:** Vaccination status of the study population

	*N*	Median age in years (IQR)	Female, *n* (%)	Median days since vaccination (IQR)^*c*^
Vaccinated in 2014/15	69	70 (66–74.5)	30 (43.5%)	101 (73.5–132.0)
Unvaccinated in 2014/15	31	66 (63–70)	13 (41.9%)	N/A
*P*-value		**0.004** ^*a*^	0.885^*b*^	

^*a*^ Mann-Whitney U. Boldface indicates median age was significantly higher in vaccinated individuals than in unvaccinated individuals.

^*b*^ Pearson χ.

^*c*^ Median days since vaccination in the 2014/15 season; N/A, not applicable.

### Effect of influenza virus infection and vaccination on antibody titers in influenza virus-infected older adults

**Fig 1:**
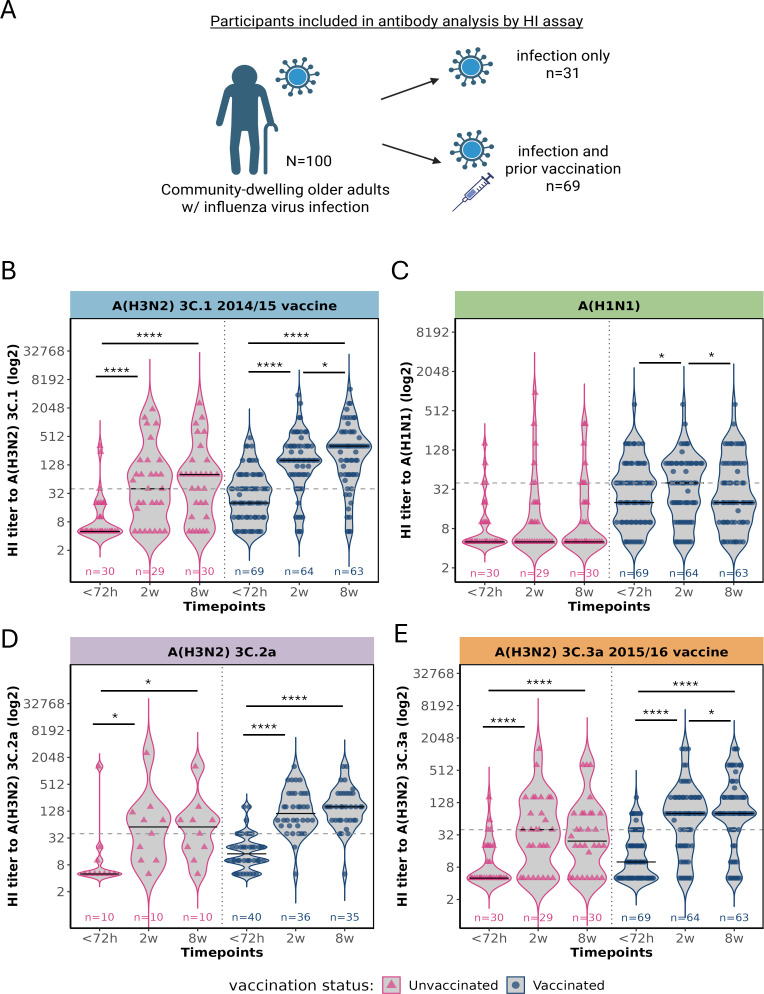
The effect of influenza virus infection on longitudinal HI titers in a cohort of influenza virus-infected older adults, stratified by vaccination status, independent of the infection strain. (**A**) Schematic overview of the samples used in panels B–E. In this figure, the HI titers against (**B**) the 2014/15 A(H3N2) and (**C**) A(H1N1) vaccine strains, (**D**) the circulating A(H3N2) strain 3C.2a, and (**E**) the 2015/16 A(H3N2) vaccine strain (3C.3a clade) are depicted in older individuals with an influenza virus infection comparing vaccinated (blue) to unvaccinated (pink) individuals across three different timepoints. For panels A, B, and D, in total, *n* = 99, *n* = 93, and *n* = 93 samples were used at timepoints <72 h, 2 weeks, and 8 weeks, respectively. For panel C, in total, *n* = 50, *n* = 46, and *n* = 45 were used for timepoints <72 h, 2 weeks, and 8 weeks, respectively. Medians are indicated by the black line. The gray dashed line corresponds to a HI titer of 40. *P*-values as determined by Wilcoxon signed-rank test: *P < 0.05; **P < 0.01; ***P < 0.001; ****P < 0.0001. Panel A was created with BioRender.com.

In order to assess whether older adults are capable of generating protective antibody responses after influenza virus infection, longitudinal antibody titers were measured by HI assay on serum samples of older adults with an influenza virus infection (Fig. 1A). In addition, the effect of vaccination on HI titers was determined by comparing individuals with an infection alone and individuals with infection and prior vaccination that season. HI titers were measured for a total of 100 individuals at timepoints <72 h, 2 weeks, and 8 weeks after infection. First, antibody titers against the A(H3N2) 3C.1 and A(H1N1) vaccine strains were evaluated using the strains that were included in the 2014/15 vaccine (Fig. 1B and C). Two weeks after infection, there was an increase in antibody titers against the A(H3N2) 3C.1 vaccine strain in influenza virus-infected individuals, regardless of vaccination status (Fig. 1B; Table S3). In contrast, the antibody titers against the A(H1N1) vaccine strain did not significantly increase over time in the majority of individuals with an infection alone, which could be due to the low number of A(H1N1) infections in the cohort (*n* = 8; Fig. 1C; Table S3). Compared with individuals with only an infection, antibody titers against both the A(H3N2) 3C.1 and the A(H1N1) vaccine strain were significantly higher in vaccinated individuals, and this was the case across all timepoints (Fig. 1B and C; Table S4).

Next, the HI titer to the main circulating A(H3N2) virus strain was assessed, belonging to HA genetic clade 3C.2a, that differed from the A(H3N2) 3C.1 2014/15 vaccine strain (Fig. 1D). Similar to the antibody responses against the A(H3N2) 3C.1 vaccine strain, the HI titers against circulating A(H3N2) mismatched strain A(H3N2) 3C.2a increased significantly 2 weeks after infection in influenza virus-infected individuals, regardless of their vaccination status (Table S3). This implies cross-reactivity of antibodies directed to the A(H3N2) 3C.1 vaccine strain and the antigenically different circulating strain A(H3N2) 3C.2a. Moreover, HI titers against the A(H3N2) 3C.3a strain, which was antigenically similar to A(H3N2) 3C.2a and included in the vaccine for the 2015/16 season, showed similar dynamics with increased titers 2 weeks after infection, further confirming antibody cross-reactivity among influenza virus strains (Fig. 1E; Table S3). Although less pronounced than seen with the 2014/15 vaccine strains, antibody titers to the circulating A(H3N2) 3C.2a strain and to the 2015/16 A(H3N2) 3C.1 vaccine strain were again higher in the vaccinated group as compared to those with an infection alone however, this did not reach significance at all timepoints (Fig. 1D and E; Table S4). Correlation analysis was performed within the vaccinated individuals in order to assess the effect of days since vaccination on the HI titers to the different influenza virus strains. HI titers showed negative correlations with the number of days since vaccination. Thus, more days since vaccination resulted in lower antibody titers, this effect reached significance for strains A(H3N2) 3C.1 and A(H3N2) 3C.3a (Fig. S1). All in all, the data suggest that, although infection alone is capable of inducing influenza virus-specific antibody responses in community-dwelling older adults, previous vaccination results in an enhanced antibody response after infection.

### Effect of infection strain on antibody responses

**Fig 2:**
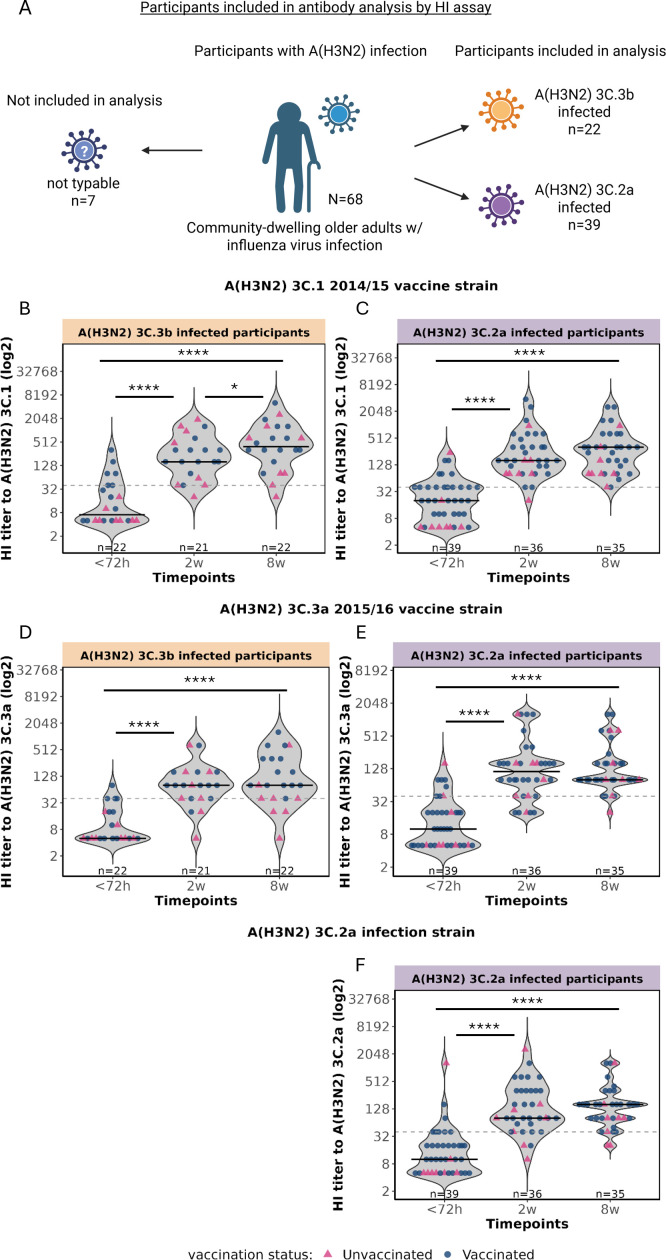
The effect of infection strain on HI titers against the vaccination and circulating strains. In these figures, (**A**) schematic overview of the samples used in panels B–F. In these figures, the HI titers against (**B and C**) the 2014/15 A(H3N2) 3C.1 vaccine strain, the (**D and E**) 2015/16 vaccine A(H3N2) 3C.3a strain, and the (**F**) circulating A(H3N2) 3C.2a strain are depicted in individuals with a A(H3N2) 3C.3b infection (B&D) and a A(H3N2) 3C.2a infection (**C, E, and F**) across three timepoints. Medians are indicated by the black line. The gray dashed line indicates an HI titer of 40. Unvaccinated individuals are indicated in pink triangles. *P*-values as determined by Wilcoxon signed rank test: *P < 0.05; **P < 0.01; ***P < 0.001; ****P < 0.0001. Panel A was created with BioRender.com.

Next, the effect of the individuals’ infection strain on the specific antibody titers was determined. Titers against the vaccine strains and circulating strains were assessed in individuals with an A(H3N2) infection, since this was the dominant infection strain (*N* = 68; Fig. 2). Infection with 3C.3b, resulted in a significant increase in antibody titers against the 2014/15 A(H3N2) vaccine strain (Fig. 2B; Table S5). Moreover, the majority of individuals had an antibody titer considered to be protective (≥40) 2 weeks after infection, and this was maintained at 8 weeks. Despite the strain mismatch, HI titers to the 2014/15 A(H3N2) vaccine strain in individuals infected with the 3C.2a strain also significantly increased over time and reached protective titers in the majority of individuals (Fig. 2C; Table S5). These data suggest cross-reactivity between the antibody responses to different A(H3N2) strains. Similar dynamics were observed when evaluating HI titers to the 2015/16 A(H3N2) vaccine strain in individuals infected with either 3C.3b or 3C.2a strain, there was a significant increase after 2 weeks and almost all individuals reached protective antibody titers (Fig. 2D and E; Table S5). In addition, HI titers against the 3C.2a infection strain in individuals infected with that strain significantly increased over time and almost all individuals reached protective antibody titers to the infection virus strain (Fig. 2F; Table S5). Comparably, although the number of A(H1N1)-infected individuals was low (*n* = 8), infection with A(H1N1) showed a trend toward higher antibody titers against the A(H1N1) vaccine strain over time (Table S5) and almost all individuals generated HI titers of 40 or above (Fig. S2A).

The antibody titers against all A(H3N2) strains significantly increased after infection, regardless of vaccination status, although some effect of vaccination was noted. Among the 3C.3b-infected individuals, significantly higher antibody titers against the 2015/16 A(H3N2) vaccine strain were observed at week 8 in individuals that received prior vaccination as compared with unvaccinated individuals (Table S6). Similarly, within the group of 3C.2a-infected individuals, vaccinated individuals exhibited significantly higher antibody titers against the 2014/15 A(H3N2) 3C.1 vaccine strain 72 h after infection (Table S6) as compared with individuals that were infected but not vaccinated. Moreover, antibody titers to A(H1N1) in both 3C.3b- and 3C.2a-infected individuals were significantly higher in vaccinated individuals at all timepoints (Fig. S1B; Table S6). Overall, the results show that influenza virus infection not only induces responses to the infection strain but also to different A(H3N2) vaccine strains, suggesting that there is functional cross-reactivity of the antibody responses between A(H3N2) strains. Not surprisingly, the antibody titers to the A(H1N1) vaccine strain in individuals infected with either A(H3N2) 3C.2a or A(H3N2) 3C.3b were significantly lower compared to their respective infection strain titers, confirming a lower grade of cross-reactivity between antibodies against A(H3N2) and A(H1N1) (Fig. S2B and C).

### Longitudinal influenza virus-specific T-cell responses in infected individuals and the effect of vaccination

**Fig 3:**
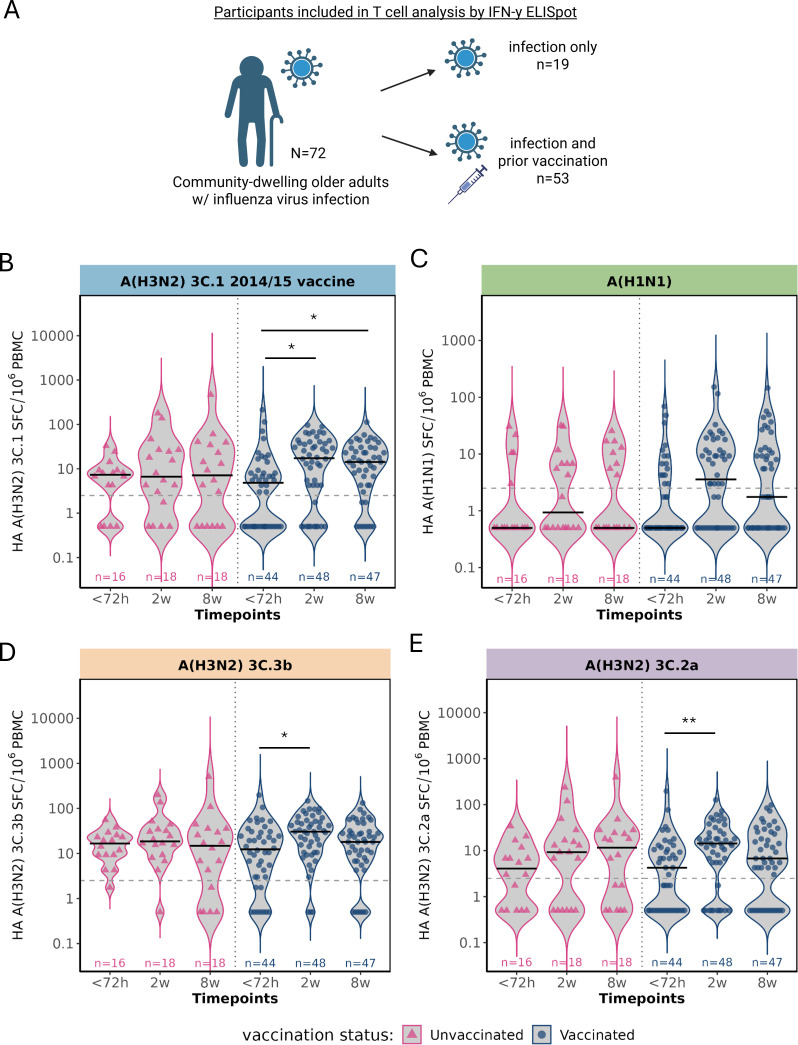
The effect of infection on longitudinal influenza virus-specific T-cell responses stratified by vaccination status. (**A**) Schematic overview of the samples used in panels B–E. The T-cell response as determined by IFN-y ELISpot is depicted. The IFN-y response after PBMC stimulation with influenza virus peptide pools of hemagglutinin (HA) of (**B**) the A(H3N2) and (**C**) A(H1N1) 2014/15 vaccine strains and the circulating strains (**D**) A(H3N2) 3C.3b, and (**E**) A(H3N2) 3C.2a is depicted in individuals with an influenza virus infection comparing vaccinated (blue) versus unvaccinated (pink) individuals. Total samples used per time point were, *n* = 60, *n* = 66, and *n* = 65 for timepoints <72 h, 2 weeks, or 8 weeks after infection respectively. Medians are indicated by the black line. The dashed line indicates a response of 2.5 SFC/10^6^ PBMC. *P*-values as determined by Wilcoxon signed-rank test: *P < 0.05; **P < 0.01; ***P < 0.001; ****P < 0.0001. Panel A was created with BioRender.com.

Next, we investigated whether influenza virus-specific T-cell responses are generated in older adults after influenza A virus infection. In addition, the effect of vaccination on these T-cell responses was assessed by comparing individuals with an infection alone and individuals with infection after previous vaccination. In a subset of the participants that had a PBMC sample available (*n* = 72; Fig. 3A), cells were stimulated with overlapping peptide pools of HA and NA of the A(H3N2) and A(H1N1) vaccine strains and the two main A(H3N2) circulating strains A(H3N2)3C.3b and A(H3N2) 3C.2a (for the latter only an HA peptide pool was measured). Antigen-specific T-cell responses were then measured by IFN-γ ELISpot using these overlapping peptide pools. The subset showed similar properties in regard to age and sex distributions as the whole population (Tables S7 and S8). In people with only an influenza virus infection, the T-cell response to HA from the A(H3N2) 3C.1 2014/15 vaccine strain appeared to slightly increase after 8 weeks (*P* = 0.055; Fig. 3B), however this trend did not remain after multiple testing correction (Table S9). In contrast, the T-cell response to the A(H1N1) vaccine strain remained low over time after infection with influenza virus (Fig. 3C). Similarly, T-cell responses to the circulating A(H3N2) 3C.3b strain showed no significant changes in individuals with an infection alone; however, most of the individuals were capable of inducing a T-cell response within 72 h after infection (>2.5 SFC/PBMC; Fig. 3D). Furthermore, the T-cell responses to the circulating A(H3N2) 3C.2a strain appear to increase 2 and 8 weeks after infection, although this did not reach significance (Fig. 3E; Table S9). These data suggest that the majority of unvaccinated older adults are capable of inducing T-cell responses within 72 h after infection with influenza virus and that there might be some degree of cross-reactivity between strain-specific T-cell responses.

When assessing the effect of vaccination on T-cell responses, it was observed that, at all timepoints, responses against the HA peptide pools of all the strains tested were comparable between vaccinated individuals and individuals with an infection alone (Table S10). However, when comparing 72 h to 2 weeks after infection, vaccinated individuals showed a significant increase in T-cell responses against HA of all A(H3N2) strains (Fig. 3B, D and E; Table S9). In addition, a trend toward higher responses against A(H1N1) was observed in vaccinated individuals, this effect did not remain after multiple testing correction (Table S9). This underlines that although vaccination did not prevent influenza virus infection, it does appear to enhance infection-induced T-cell responses, with cross-reactive potential. In addition, we tested T-cell responses against the highly conserved matrix protein M1. The T-cell responses to the peptide pool of internal protein M1 significantly increased in the vaccinated group 2 weeks after infection but not in individuals with an infection alone (Fig. S3; Table S9). In addition to responses to HA, the T-cell responses to neuraminidase (NA) of the two vaccine strains and the A(H3N2) 3C.3b strain were measured. Overall, responses to NA appeared to show similar dynamics compared to HA, with increased T-cell responses over time after infection, with stronger increases in vaccinated individuals (Fig. S4). In contrast to the antibody titers, the number of days between vaccination and the peak of T-cell responses were not correlated for any of the strains (Fig. S5). All in all, strain-specific T-cell responses appear to be induced in older adults with influenza virus infection, with a stronger increase of influenza virus-specific T-cell responses observed in influenza virus-infected individuals that were vaccinated before infection. However, the apparent effect of vaccination on T-cell responses is less pronounced than observed with the antibody response.

### Effect of infection strain on influenza virus-specific T-cell responses

**Fig 4:**
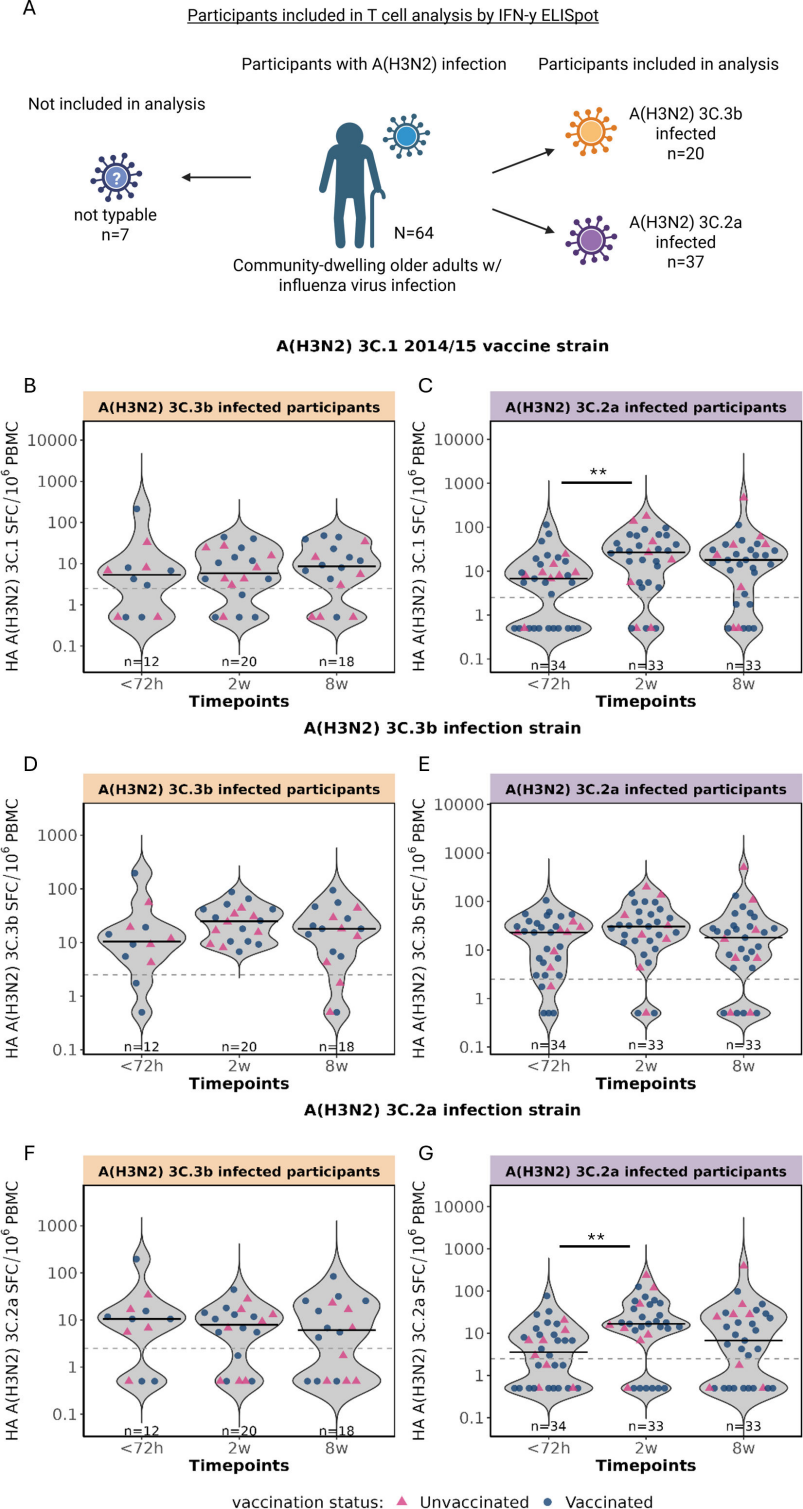
Influenza virus-specific T-cell responses to vaccine and infection strains in older adults with influenza virus infection. (**A**) Schematic overview of the samples used in panels B-G. The T-cell responses against the (**B and C**) A(H3N2) vaccine strain (3C.1) and (**D–G**) A(H3N2) circulating strains (3C.3b and 3C.2a) are depicted in individuals infected with A(H3N2) 3C.3b (**B, D, and F**) and A(H3N2) 3C.2a (**C, E, and G**). Individuals unvaccinated in 2014/15 are shown in pink, and individuals vaccinated in 2014/15 are shown in blue. The dashed line indicates a response of 2.5 SFC/10^6^ PBMC. Medians are indicated by the black line. *P*-values as determined by Wilcoxon signed-rank test: *P < 0.05; **P < 0.01; ***P < 0.001; ****P < 0.0001. Panel A was created with BioRender.com.

Finally, the effect of the individuals’ infection strain on T-cell responses to both vaccine and infection strains was analyzed (Fig. 4). As with the antibody titer assessment, T-cell responses against the vaccine strains and circulating strains were assessed in individuals with an A(H3N2) infection, since this was the dominant infection strain. In individuals with an A(H3N2) 3C.3b infection, the response to peptide pools of the A(H3N2) vaccine strain and the A(H3N2) 3C.3b infection strain did not significantly increase over time (Fig. 4B and D). However, almost all individuals that were infected with an A(H3N2) 3C.3b strain already generated a T-cell response (>2.5 SFC/ PBMC) to the 3C.3b strain within 72 h after infection, suggesting that there may already have been a memory response present to A(H3N2) 3C.3b or an antigenically similar strain. After infection with the A(H3N2) 3C.2a strain, individuals mounted significantly higher T-cell responses to the A(H3N2) 3C.1 vaccine strain as well as to their infection strain when comparing 72 h to 2 weeks after infection (Fig. 4C and G; Table S11). Moreover, the response to the vaccine strain showed a trend toward increased response after 8 weeks. Results show that, in individuals infected with 3C.2a, there is not only a T-cell response to the infection strain but also to the A(H3N2) 3C.1 vaccine strain, implying that there is cross-reactivity of T-cell responses between these strains. Although it did not reach significance, most individuals infected with A(H3N2) 3C.2a appeared to be able to induce T-cell responses to the A(H3N2) 3C.3b strain over time (Fig. 4E). In contrast, individuals infected with A(H3N2) 3C.3b did not show an increase in T-cell responses to the A(H3N2) 3C.2a strain (Fig. 4F; Table S11). This indicates that individuals infected with A(H3N2) 3C.2a may have pre-existing immunity to A(H3N2) 3C.3b due to its antigenic similarity to the A(H3N2) 2014/15 vaccine strain (3C.1 clade), but A(H3N2) 3C.3b-infected individuals have less pre-existing cross-reactive immunity to A(H3N2) 3C.2a since this strain was an antigenic drift variant.

**Fig 5:**
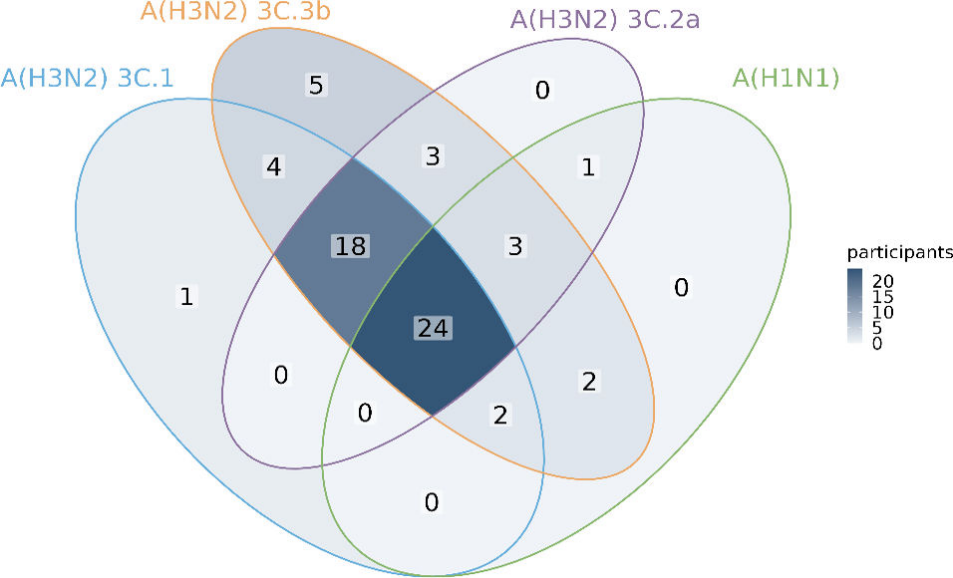
Number of individuals with T-cell response to at least one influenza A strain. Venn diagram depicting the overlap in T-cell responses against the four different influenza A virus strains. Individuals with a T-cell response against at least one strain at the 2-week timepoint were included in this figure (*n* = 63). T-cell response was defined as having >2.5 SFC/10^6^ PBMC.

Additionally, most individuals infected with an A(H1N1) strain were able to mount a T-cell response to the A(H1N1) strain after 2 weeks (Fig. S6A). In contrast, responses to the A(H1N1) strain in individuals with A(H3N2) infections, either A(H3N2) 3C.2a or A(H3N2) 3C.3b, were lower compared with the response to their infection strain and did not show significant increases over time (Fig. S6B; Table S11). Responses to NA of the A(H3N2) vaccine strain and the circulating A(H3N2) 3C.3b strain in individuals with an A(H3N2) 3C.3b or A(H3N2) 3C.2a infection showed similar dynamics compared with HA (Fig. S7A through D). Correspondingly, responses to NA of the A(H1N1) vaccine strain in individuals with A(H1N1) infections were also comparable to HA responses (Fig. S7E). There was no clear effect of vaccination on the T-cell responses against HA within the A(H3N2) 3C.3b- and A(H3N2) 3C.2a-infected individuals (Table S12). Although less prominent than the HI antibody data, the influenza virus-specific T-cell responses appear to show cross-reactivity between vaccination and infection strains. Potential cross-reactivity of T-cell responses between the different influenza A strains was further illustrated by a Venn diagram. Within the group of individuals that show a T-cell response to at least one strain (*n* = 63/66) at the peak of T-cell responses (2 weeks after influenza infection), the majority (*n* = 42) showed responses to either all influenza A strains (*n* = 24/63) or to all the A(H3N2) strains (*n* = 18/63; Fig. 5). These data further suggest that there is cross-reactivity of the virus-specific T-cell responses between the influenza A strains tested.

### Correlation analyses between influenza virus-specific immune responses, vaccination status, and symptom burden

Next, we assessed whether there was a relationship between the influenza virus-specific antibody response and the influenza virus-specific T-cell responses. No correlations between HI titers and IFN-γ responses were found for any of the strains at any of the timepoints, as determined by Spearman correlation (Fig. S8).

In addition, in order to investigate the relation between the influenza virus strain-specific immune responses and symptom burden after influenza virus infection, we assessed the correlation between HI titers and individual-level symptom outcomes as well as T-cell responses and symptom outcomes. The number of symptoms, symptom duration, as well as fever duration did not show significant correlations to either HI titers or IFN-γ levels 2 weeks after infection after multiple testing correction (Tables S13 and S14). Furthermore, we assessed whether vaccination status had an effect on the symptom outcomes. When comparing the number of symptoms, duration of symptoms, and the duration of fever, between vaccinated and unvaccinated individuals, no significant differences were found. There appeared to be a trend toward decreased fever duration in vaccinated individuals after initial analysis; however, this effect did not remain after multiple testing correction (Table S15).

## DISCUSSION

In this study, the longitudinal influenza virus-specific antibody and T-cell responses were described for a cohort of community-dwelling older adults in the Netherlands after influenza virus infection. In addition, the effect of prior influenza vaccination on both the humoral and the T-cell response was assessed at several timepoints, as well as the effect of infection strain on these responses. Furthermore, the potential cross-reactivity of influenza virus-specific antibody and T-cell responses between the different vaccine and circulating strains was evaluated. We found that older individuals with an influenza virus infection were capable of inducing antibody responses to different influenza virus strains, regardless of whether they had received an influenza vaccination in that season. However, prior influenza vaccination did result in higher titers after infection as compared with individuals with only an influenza virus infection. In the T-cell compartment, there was a significant increase in responses to A(H3N2) strains in vaccinated individuals when comparing 72 h after infection to 2 weeks after infection, but responses were not significantly higher compared to individuals with an influenza virus infection alone. Despite the mismatch between vaccine and circulating A(H3N2) strains in the 2014/15 season, individuals were able to generate antibodies and T-cell responses to both, strongly indicating cross-reactivity of these responses.

This study was performed in the influenza season of 2014/15 in the Netherlands, a season that was hallmarked by low vaccine effectiveness due to a vaccine mismatch. Our findings showed that older adults with an influenza virus infection elicited antibody responses to the 2014/15 A(H3N2) vaccine strain (3C.1 clade) as well as the antigenically drifted 2015/16 A(H3N2) vaccine strain (3C.3a clade) and circulating strain A(H3N2) 3C.2a, irrespective of vaccination status. These dynamics were also demonstrated when analyzing A(H3N2) clade 3C.3b and clade 3C.2a infected individuals separately. The results suggest that there are antibodies present in older adults that are capable of targeting multiple different strains, despite antigenic drift. This cross-reactivity was also observed in the influenza virus-specific T-cell responses, where strain-specific T-cell responses were mounted to peptide pools of HA from the 2014/15 A(H3N2) vaccine strain as well as the two main circulating A(H3N2) strains (3C.2a and 3C.3b), albeit less pronounced compared with the antibody response. In a previous study performed on children and adolescents in the 2014/15 season, cross-reactivity of antibodies after vaccination was also observed, and this was linked to age and pre-existing immunity from prior exposure (38). Older adults have lifelong infection and vaccination histories for influenza virus that shape the B-cell repertoire and memory T-cell responses that can influence the ability to generate cross-reactive responses (39–42). Adults generally have a broader antibody response after natural infection, they can induce antibodies targeting historic influenza virus strains encountered earlier in life, as well as the infection strain, and these responses are often long-lived (43). However, these recall responses are usually more often directed against strains that are antigenically more similar.

Although the circulating A(H3N2) 3C.2a strain in 2014/15 showed antigenic drift from the vaccine strain, our data indicate that this had not led to the complete absence of cross-reactive HI antibody responses, which can be explained by shared epitopes on the head of HA that leads to partial cross-reactivity. In addition, in our study, majority of individuals were capable of mounting T-cell responses to all A(H3N2) strains 2 weeks after influenza virus infection, further indicating cross-reactivity between T-cell responses against these strains. In previous studies, pre-existing T-cell responses have been associated with protection against influenza disease (42, 44, 45). In contrast to the antibody response, natural infection with influenza virus results in T-cell responses that are generated to more conserved parts of the virus, such as the stalk of HA or internal influenza virus proteins, such as M1, allowing for broader recognition between different subtypes that might have drifted from each other (42, 46). Because of the observed cross-reactive T-cell response to conserved regions of the virus and their capability to clear infection, the development of a universal influenza vaccine is highly focused on inducing cross-reactive T-cell responses, which could evade the high mutation rate of influenza viruses (47, 48).

Current influenza vaccines are mainly designed to induce antibodies against HA. In correspondence with that, in our study population, vaccination further boosted the antibody responses after natural infection and led to significantly higher HI antibody titers compared to unvaccinated individuals. On a population scale, serum antibody titers of 40 or higher are correlated with a 50% decrease in risk for influenza virus infection and disease (49). In line with that, although we observed that most individuals in this study reached antibody titers of ≥40 to their infection strain, regardless of whether they were vaccinated or not, they were not completely protected from infection or developing symptoms. Furthermore, previous studies have shown that antibody titers are a poor correlate of protection in older adults especially, also underscoring the importance of including T-cell analyses (12, 13).

Besides the antibody response, cell-mediated immunity is pivotal for clearing the virus from infected cells whenever antibodies fail to provide protection. Indeed, cross-protective immunity in the mucosa has previously been associated with presence of cytotoxic CD8 T-cell responses (50). Older adults depend more heavily on the recall of these previously formed cross-reactive T-cell responses since current vaccines are not designed to induce T-cell responses and thus only weakly stimulate virus-specific CD8 T-cells (46). In this study, we found that the influenza virus-specific T-cell response to HA of several strains was comparable between vaccinated and unvaccinated individuals at all time points after infection. However, vaccinated individuals did appear to show significantly higher T-cell responses 2 weeks after infection, confirming that vaccination does have some impact on the T-cell response, potentially by recall of recent memory responses formed after vaccination. In addition to the significant increase in T-cell responses to HA of different A(H3N2) strains, we observed that in vaccinated individuals, the response to M1 was also significantly increased two weeks after infection. M1 protein is not actively included in vaccines; however, contaminations in the vaccine with M1 as well as previous infection with influenza virus could have generated memory responses to this internal influenza virus protein, which is similar across A(H3N2) and A(H1N1) subtypes (46). The vaccinated group was significantly older and may have formed stronger memory T-cell responses due to multiple exposures to genetically similar influenza viruses (51).

Despite vaccination and the presence of immune responses against influenza virus, the individuals in our study were still infected. However, vaccinated individuals did show a trend toward less days with fever compared to unvaccinated individuals. Although this trend did not remain after multiple testing correction, it could indicate that vaccination might still have provided some protection. Indeed, studies on vaccination and symptom severity showed that disease severity is attenuated in vaccinated individuals (52, 53), with fever being the most commonly reduced symptom (52).

A limitation of this study is that antibodies were measured by HI assay, which mainly detects antibodies targeting the head of HA. Antibodies targeting more conserved regions on HA, such as the stalk, or non-neutralizing antibodies are not detected in this assay (39, 54), thus not fully representing the whole range of antibodies that are produced after influenza virus infection. The T-cell responses in this study were determined in PBMC by IFN-y ELISpot, although this is common practice (55), it does have its limitations. Other cells that are present in the PBMC fraction, such as NK cells, may produce IFN-y in this type of assay and therefore the readout theoretically could contain more than solely T-cell responses (56). In addition, with the current set-up we are unable to distinguish between CD4 and CD8 T-cell responses, separation of CD4 and CD8 T-cells before peptide stimulation could have enabled a deeper understanding of which T cells exactly are predominantly responsible for the IFN-y response.

In line with what was observed on a population scale in the 2014/15 season in the Netherlands (32), the majority of individuals in this study were infected with an A(H3N2) influenza virus strain. This is a strain that is known for causing a more severe disease course with increased morbidity and mortality in older adults (46, 57). However, in contrast with the excess mortality and high disease burden observed in the general population, in this study group no severe disease, hospitalizations, or deaths were noted (data not shown), and infection-induced antibody as well as T-cell responses in most of the individuals. This discrepancy could possibly be due to the study set-up where we acquired samples from generally healthy community-dwelling older adults (34, 36), and thus not including the more frail institutionalized older individuals that generally have a higher disease burden (46). In addition, it is possible that the presence of cross-reactive immune responses, at least in part, contributed to the low frequency of severe illness in our study population. Indeed, as mentioned before, pre-existing cross-reactive T-cells against conserved epitopes have been associated with less severe influenza illness (20, 42, 45, 58). Further underscoring the importance of such responses in protection against influenza. Furthermore, the relatively homogeneous population in terms of disease severeness might also have contributed to the absence of correlations found between the immune responses and the number of symptoms, symptom duration, or fever duration. In conclusion, despite the older age and the potential immunosenescence that comes with it, the individuals in this study group were capable of mounting HI antibody titers and T-cell responses after infection with influenza virus. Moreover, despite a mismatch between A(H3N2) strains of vaccine and circulating strains in the 2014/15 season, the generated antibody and T-cell responses appeared to show cross-reactivity. The extensive infection and vaccination history of older adults makes investigating the cross-reactive adaptive immune responses against influenza virus very complex. Further elucidating the exact mechanisms behind cross-reactive immune responses and how infection and vaccination history impacts these responses would be highly beneficial for the development of new vaccines. Ideally, efforts to design new vaccine strategies should focus on enhancing breath and durability of both cross-reactive antibody and T-cell responses to influenza virus to prevent disease, especially in the vulnerable aging population.

## MATERIALS AND METHODS

### Study cohort

Humoral and cellular responses were assessed in older adults presenting with influenza-like illness in the “Identification of potential pathogens responsible for influenza-like illness and evaluation of humoral and cellular immunity against identified microorganisms in elderly in The Netherlands” (ILI-3) study. In the ILI-3 study, adults 60 years and older were followed during the influenza season of 2014/15 in order to monitor the contribution of influenza virus to influenza-like illness (ILI) in the Netherlands. The results and design of the study have previously been described (34, 36). In brief, participants were asked to monitor and report ILI events according to the Dutch pel criteria as defined by fever (≥ 37.8°C) and at least one other symptom, including headache, muscle pain, coughing, rhinitis, chest pain, and/or sore throat (34, 37). In case of an ILI event, a home visit was conducted by a study nurse within 72 h after fever onset as well as 2–3 weeks post-ILI and at 7–8 weeks (±1 week) post-ILI (“recovery visit”). Fever onset was considered to be the start of infection. The number of symptoms, symptom duration (in days), and fever duration (in days) were all documented. At all visits, pharyngeal swabs, serum, and PBMC samples were collected and processed, as previously described (36). For the study described here, only individuals with a confirmed influenza virus infection and available serum and PBMC samples were included in the analyses.

### Influenza virus (sub)typing

Influenza virus infection was detected by analysis of RNA isolated from nasopharyngeal and oropharyngeal swabs using multiplex ligation-dependent probe amplification (MLPA) assay (RespiFinder Smart 22 kit; Pathofinder, the Netherlands), as described before (36). Samples that were tested positive for influenza virus were further subtyped by real-time RT-PCR using the Roche Lightcycler 480 system with a slightly amended protocol (59) (60). Phylogenetic clades of the influenza A (H3N2) viruses were determined by Sanger sequencing of hemagglutinin (HA) using universal influenza virus type A primers, as described previously (34).

### Hemagglutination inhibition (HI) assay

Influenza virus-specific antibody titers were assessed by HI assay according to the standard protocol of the World Health Organization (61). In brief, twofold serial dilutions were made of serum samples and incubated with influenza virus followed byincubation with turkey erythrocytes. Antibody titers preventing agglutination were calculated from scoring the agglutination of turkey erythrocytes. HI titers were determined against four influenza A virus strains on serum samples of influenza virus-infected individuals at <72 h, 2 weeks, and 8 weeks after infection (respectively *n* = 99, *n* = 93, and *n* = 93 out of *n* = 100). The following vaccine strains were evaluated: A/California/07/2009 (H1N1) and A/Texas/50/2012 (H3N2) (3C.1 clade) from the 2014/15 vaccine and the 2015/16 vaccine strain A/Switzerland/9,715,293/2013 (H3N2) (3C.3a clade). Due to lack of suitable 3C.2a vaccine candidates at the time, recommendations were made to replace the 3C.1 vaccine strain with a 3C.3a strain for the 2015/16 influenza season vaccine because this strain was antigenically indistinguishable from 3C.2a in HI assays (35, 62). In addition, the main A(H3N2) circulating strain A/Hongkong/4801/2014 (3C.2a clade) (H3N2) was evaluated. HI titers against the 3C.2a strain were only determined in a subset of individuals (*n* = 50, *n* = 46, and *n* = 45). All virus strains were supplied by NIBSC (United Kingdom). HI titers of 40 or higher were considered to be protective. For the statistical analyses, the 2-log transformed data were used.

### IFN-γ ELISpot

IFN-γ enzyme-linked immunospot (ELISpot) assay was performed on a subset of influenza virus-infected individuals that had a PBMC sample available (*n* = 72) for at least one of the study timepoints: <72 h, 2 weeks, or 8 weeks after infection (respectively *n* = 60, *n* = 66 and *n* = 65 out of *n* = 72). PBMCs were stimulated with 15-mer peptide pools with 11 amino acid overlap (JPT Peptide Technologies GmbH, Germany) covering hemagglutinin (HA) protein and Neuraminidase (NA) protein from vaccine strains A/Texas/50/2012 (H3N2) and A/California/07/2009 (H1N1), and circulating strain A/Netherlands/525/2014 (H3N2) (3C.3b clade). For the circulating A/Hongkong/4801/2014 (H3N2) (3C.2a clade) strain, only an HA peptide pool was available. In addition to the HA and NA peptide pools, IFN-γ responses were also measured against an influenza virus M1 peptide pool. All peptide pools were used at a concentration of 1 µg/mL. Per well, 400,000 PBMCs were stimulated and incubated for 18 h at 37°C on 96-well membrane-bottom plates (PVDF plate MSIPS4510, Millipore) coated with anti-IFN-γ (Mabtech AB, Sweden). Plates were then incubated with biotin-labeled IFN-γ antibody for 2 h at room temperature, followed by washing with PBS. Subsequently, plates were incubated with streptavidin-alkaline phosphatase (ALP) in PBS with 0.5% fetal calf serum (FCS) for 1 h at room temperature. After washing, substrate was added to the plates. Color reactions were stopped by adding tap water, and plates were dried in the incubator at 60°C for 30 min. Spots were counted on the A.EL.VIS reader (A.EL.VIS GmbH, Germany). Results are reported as spot-forming cells (SFCs) per 10^6^ PBMCs and are the mean of duplicate measurements. Measurements were corrected for background responses by subtracting the negative control. If no response to the positive control with SEB was observed or response to SEB was <50 spots, samples were excluded from further analysis. A constant value of 0.5 was added to all data points after sample exclusion. SFC ≥ 2.5/10^6^ PBMCs was defined as a response.

### Statistical analyses

Statistical analyses were performed in IBM SPSS Statistics version 29 (IBM Corporation, Armonk, NY, USA) with *P*-values of ≤ 0.05 considered statistically significant. For each influenza strain, we performed pairwise comparisons between timepoints using the Wilcoxon signed-rank test, followed by multiple testing correction (Holm-Bonferroni), which was applied within each strain. Unpaired data were analyzed by Mann-Whitney U test, again followed by Holm-Bonferroni multiple testing correction. Correlation analyses were performed using the Spearman test. Frequency analysis by χ test or Fisher’s exact test, as well as Spearman correlations, was determined using GraphPad Prism (v. 10.6.1). Figures were generated in RStudio (v4.4.1) using the Tidyverse package (63). The schematics in Fig. 1A, 2A, 3A and 4A were created with BioRender.com.

## Data Availability

The data supporting the findings in this paper can be made available upon reasonable request.

## SUPPLEMENTAL MATERIAL

Supplemental material jvi.00407-26-s0001.pdf Tables S1 to S15; Fig. S1 to S8.

ASM does not own the copyrights to Supplemental Material that may be linked to, or accessed through, an article. The authors have granted ASM a non-exclusive, world-wide license to publish the Supplemental Material files. Please contact the corresponding author directly for reuse.
