# Supplementary material for: Cross-reactive antibody and T-cell responses after influenza virus infection in community-dwelling older adults
Source: J Virol. 2026 May 12;100(6):e00407-26. doi: 10.1128/jvi.00407-26 (PMC13288483; doi:10.1128/jvi.00407-26)
Supplement: Supplemental material — Tables S1 to S15; Fig. S1 to S8. [file jvi.00407-26-s0001.pdf]

Supplementary materials to:

**Cross-reactive antibody and T-cell responses after influenza virus infection in community-dwelling older adults**

Lisa van Pul[a], Sietske Karla Rosendahl Huber[a], Ronald Jacobi[a], Marion Hendriks[a], Roos van Schuijlenburg[a], Yannick van Sleen[a], Elske Bijvank[a], Jelle de Wit[a], Josine van Beek[a]#

[a]Centre for Infectious Disease Control, National Institute for Public Health and the Environment (RIVM), Bilthoven, The Netherlands

**Table S1. Number of vaccinations received between the 2009/10 and 2013/14 influenza seasons.**

| | Vaccinations, n | Participants, n (%) |
|---|---|---|
| **Unvaccinated** | 0 | 26 (83.9%) |
| | 1 | 2 (6.5%) |
| | 2 | 2 (6.5%) |
| | 3 | 1 (3.2%) |
| **Vaccinated** | 1 | 3 (4.3%) |
| | 2 | 1 (1.4%) |
| | 3 | 3 (4.3%) |
| | 4 | 5 (7.2%) |
| | 5 | 57 (82.6%) |

**Table S2: Number of viral co-infections in influenza virus infected individuals**

| Co-infections with influenza virus | Participants, n |
|---|---|
| **Rhinovirus** | 3 |
| **Seasonal coronaviruses** | 3 |
| **RSV** | 3 |
| **hMPV** | 0 |
| **Bocavirus** | 0 |
| **Rhinovirus - RSV** | 1 |

hMPV: human metapneumovirus; RSV: Respiratory syncytial virus

**Table S3: Wilcoxon signed rank test of HI titers against influenza virus per vaccination status comparing timepoints**

| Vaccination status | Titers against influenza virus strain | Time point Pairs tested | N pairs | Z | P-value* | Adjusted P-value ** |
|---|---|---|---|---|---|---|
| **unvaccinated** | A(H3N2) 3C.1 2014/15 vaccine strain | <72 hours vs. 2 weeks | 29 | -3.705 | **<0.001** | **<0.001** |
| | | <72 hours vs. 8 weeks | 29 | -3.591 | **<0.001** | **<0.001** |
| | | 2 weeks vs. 8 weeks | 28 | -0.063 | 0.949 | 0.949 |
| | A(H3N2) 3C.2a | <72 hours vs. 2 weeks | 10 | -2.439 | **0.015** | **0.044** |
| | | <72 hours vs. 8 weeks | 10 | -2.389 | **0.017** | **0.034** |
| | | 2 weeks vs. 8 weeks | 10 | -0.368 | 0.713 | 0.713 |
| | A(H3N2) 3C.3a 2015/16 vaccine strain | <72 hours vs. 2 weeks | 29 | -3.650 | **<0.001** | **<0.001** |
| | | <72 hours vs. 8 weeks | 29 | -3.775 | **<0.001** | **<0.001** |
| | | 2 weeks vs. 8 weeks | 28 | -1.565 | 0.118 | 0.118 |
| | A(H1N1) | <72 hours vs. 2 weeks | 29 | -1.611 | 0.107 | 0.322 |
| | | <72 hours vs. 8 weeks | 29 | -1.552 | 0.121 | 0.241 |
| | | 2 weeks vs. 8 weeks | 28 | -0.687 | 0.492 | 0.492 |
| **vaccinated** | A(H3N2) 3C.1 2014/15 vaccine strain | <72 hours vs. 2 weeks | 64 | -5.939 | **<0.001** | **<0.001** |
| | | <72 hours vs. 8 weeks | 63 | -6.161 | **<0.001** | **<0.001** |
| | | 2 weeks vs. 8 weeks | 59 | -2.212 | **0.027** | **0.027** |
| | A(H3N2) 3C.2a | <72 hours vs. 2 weeks | 36 | -5.065 | **<0.001** | **<0.001** |
| | | <72 hours vs. 8 weeks | 35 | -5.107 | **<0.001** | **<0.001** |
| | | 2 weeks vs. 8 weeks | 32 | -0.770 | 0.442 | 0.442 |
| | A(H3N2) 3C.3a 2015/16 vaccine strain | <72 hours vs. 2 weeks | 64 | -6.043 | **<0.001** | **<0.001** |
| | | <72 hours vs. 8 weeks | 63 | -6.111 | **<0.001** | **<0.001** |
| | | 2 weeks vs. 8 weeks | 59 | -2.368 | **0.018** | **0.018** |
| | A(H1N1) | <72 hours vs. 2 weeks | 64 | -2.244 | **0.025** | **0.050** |
| | | <72 hours vs. 8 weeks | 63 | -0.504 | 0.614 | 0.614 |
| | | 2 weeks vs. 8 weeks | 59 | -2.709 | **0.007** | **0.020** |

* Exact 2-tailed P-values determined by Wilcoxon signed rank test; ** Holm-Bonferroni adjusted P-values; P-values <0.05 are depicted in bold

**Table S4: Mann-Whitney U test comparing the HI titers to influenza virus between vaccinated vs. unvaccinated.**

| Titers against influenza virus strain | Time point | group | N | Mean rank | U | Z | Unadjusted P-value* | Adjusted P-value** |
|---|---|---|---|---|---|---|---|---|
| A(H3N2) 3C.1 2014/15 vaccine strain | <72 hours | vaccinated | 69 | 58.28 | 463.5 | -4.448 | **<0.001** | **<0.001** |
| | | unvaccinated | 30 | 30.95 | | | | |
| | 2 weeks | vaccinated | 64 | 52.82 | 555.5 | -3.113 | **0.002** | **0.002** |
| | | unvaccinated | 29 | 34.16 | | | | |
| | 8 weeks | vaccinated | 63 | 53.33 | 546.0 | -3.303 | **<0.001** | **0.002** |
| | | unvaccinated | 30 | 33.70 | | | | |
| A(H1N1) | <72 hours | vaccinated | 69 | 59.11 | 406.5 | -4.885 | **<0.001** | **<0.001** |
| | | unvaccinated | 30 | 29.05 | | | | |
| | 2 weeks | vaccinated | 64 | 53.98 | 481.5 | -3.763 | **<0.001** | **<0.001** |
| | | unvaccinated | 29 | 31.60 | | | | |
| | 8 weeks | vaccinated | 63 | 53.40 | 541.5 | -3.380 | **0.001** | **0.001** |
| | | unvaccinated | 30 | 33.55 | | | | |
| A(H3N2) 3C.2a | <72 hours | vaccinated | 40 | 27.81 | 107.5 | -2.314 | **0.023** | **0.046** |
| | | unvaccinated | 10 | 16.25 | | | | |
| | 2 weeks | vaccinated | 36 | 25.39 | 112.0 | -1.830 | 0.072 | 0.072 |
| | | unvaccinated | 10 | 16.70 | | | | |
| | 8 weeks | vaccinated | 35 | 25.56 | 85.5 | -2.490 | **0.013** | **0.039** |
| | | unvaccinated | 10 | 14.05 | | | | |
| A(H3N2) 3C.3a 2015/16 vaccine strain | <72 hours | vaccinated | 69 | 53.46 | 796.5 | -1.925 | 0.054 | 0.108 |
| | | unvaccinated | 30 | 42.05 | | | | |
| | 2 weeks | vaccinated | 64 | 50.53 | 702.0 | -1.899 | 0.058 | 0.058 |
| | | unvaccinated | 29 | 39.21 | | | | |
| | 8 weeks | vaccinated | 63 | 53.06 | 563.0 | -3.185 | **0.001** | **0.004** |
| | | unvaccinated | 30 | 34.27 | | | | |

* P-values determined by Mann-Whitney U test; ** Holm-Bonferroni adjusted P-values; P-values <0.05 are depicted in bold

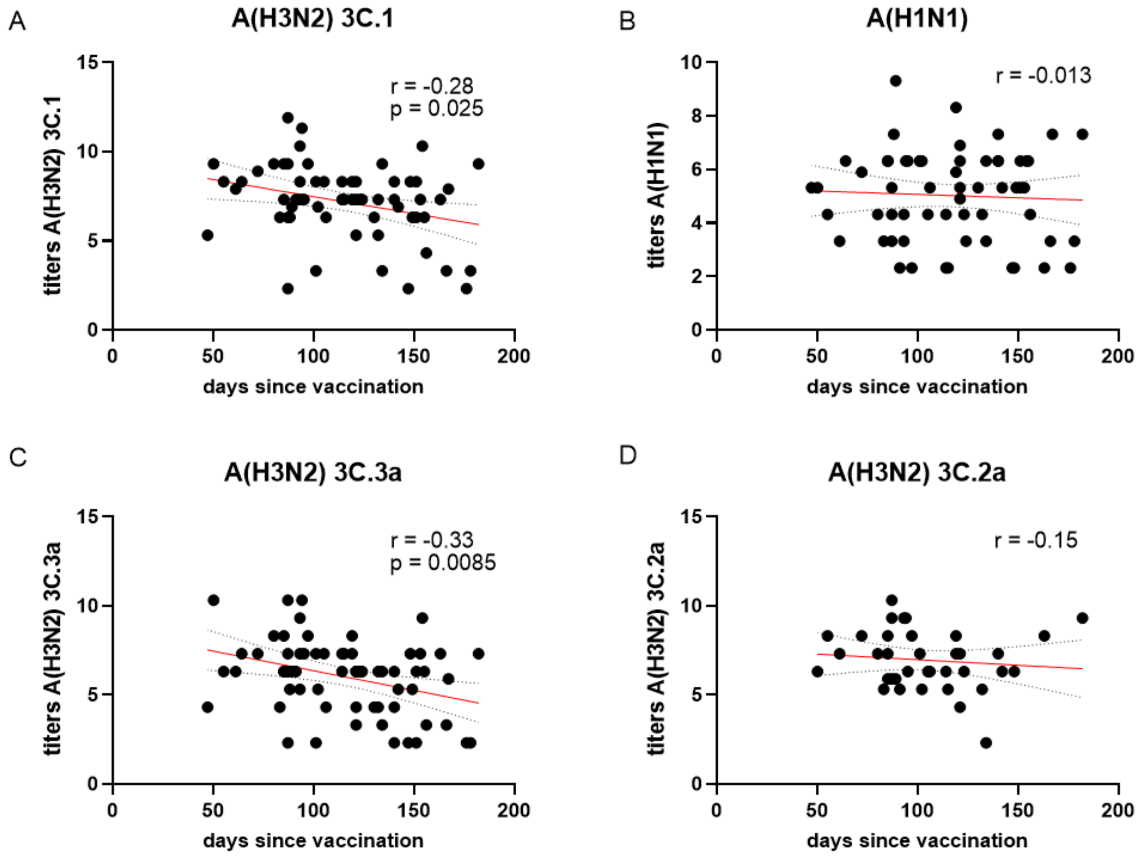


**Figure S1: Correlation between HI titers and the number of days since vaccination.** In this figure the correlations between the days since vaccination and the HI titers against the (A) 2014/15 A(H3N2) 3C.1 vaccine strain, (B) A(H1N1) strain, (C) 2015/16 A(H3N2) 3C.3a vaccine strain and the circulating A(H3N2) 3C.2a strain are depicted. Spearman r-coefficients and significant P-values are depicted in the plots

**Table S5: Wilcoxon signed rank test HI titers against influenza virus strains comparing timepoints within the infection strain**

| Infection strain | Titers against | Time point Pairs tested | N | Z | P-value* | Adjusted P-value ** |
|---|---|---|---|---|---|---|
| **A(H3N2) 3C.3b** | A(H3N2) 3C.1 2014/15 vaccine strain | <72 hours vs. 2 weeks | 21 | -3.926 | **<0.001** | **<0.001** |
| | | <72 hours vs. 8 weeks | 22 | -4.020 | **<0.001** | **<0.001** |
| | | 2 weeks vs. 8 weeks | 21 | -2.506 | **0.012** | **0.012** |
| | A(H3N2) 3C.3a 2015/16 vaccine strain | <72 hours vs. 2 weeks | 21 | -3.944 | **<0.001** | **<0.001** |
| | | <72 hours vs. 8 weeks | 22 | -4.034 | **<0.001** | **<0.001** |
| | | 2 weeks vs. 8 weeks | 21 | -1.355 | 0.175 | 0.175 |

| | | | | | | |
|---|---|---|---|---|---|---|
| | A(H1N1) | <72 hours vs. 2 weeks | 21 | -1.633 | 0.102 | 0.102 |
| | | <72 hours vs. 8 weeks | 22 | -2.333 | **0.020** | **0.039** |
| | | 2 weeks vs. 8 weeks | 21 | -2.585 | **0.010** | **0.029** |
| **A(H3N2) 3C.2a** | A(H3N2) 3C.1 14/15 vaccine strain | <72 hours vs. 2 weeks | 36 | -5.105 | **<0.001** | **<0.001** |
| | | <72 hours vs. 8 weeks | 35 | -5.184 | **<0.001** | **<0.001** |
| | | 2 weeks vs. 8 weeks | 33 | -0.799 | 0.425 | 0.425 |
| | A(H3N2) 3C.2a | <72 hours vs. 2 weeks | 36 | -5.180 | **<0.001** | **<0.001** |
| | | <72 hours vs. 8 weeks | 35 | -5.122 | **<0.001** | **<0.001** |
| | | 2 weeks vs. 8 weeks | 33 | -0.191 | 0.849 | 0.849 |
| | A(H3N2) 3C.3a 2015/16 vaccine strain | <72 hours vs. 2 weeks | 36 | -5.239 | **<0.001** | **<0.001** |
| | | <72 hours vs. 8 weeks | 35 | -5.183 | **<0.001** | **<0.001** |
| | | 2 weeks vs. 8 weeks | 33 | -0.422 | 0.673 | 0.673 |
| | A(H1N1) | <72 hours vs. 2 weeks | 36 | -0.108 | 0.914 | 0.914 |
| | | <72 hours vs. 8 weeks | 35 | -2.121 | **0.034** | 0.102 |
| | | 2 weeks vs. 8 weeks | 33 | -1.684 | 0.092 | 0.184 |
| **A(H1N1)** | A(H3N2) 3C.1 14/15 vaccine strain | <72 hours vs. 2 weeks | 8 | -1.414 | 0.157 | 0.471 |
| | | <72 hours vs. 8 weeks | 7 | -0.816 | 0.414 | 0.828 |
| | | 2 weeks vs. 8 weeks | 7 | 0.000 | 1.000 | 1.000 |
| | A(H3N2) 3C.2a | <72 hours vs. 2 weeks | 3 | -1.000 | 0.317 | 0.317 |
| | | <72 hours vs. 8 weeks | 3 | -1.414 | 0.157 | 0.471 |
| | | 2 weeks vs. 8 weeks | 3 | -1.000 | 0.317 | 0.634 |
| | A(H3N2) 3C.3a 2015/16 vaccine strain | <72 hours vs. 2 weeks | 8 | -1.633 | 0.102 | 0.306 |
| | | <72 hours vs. 8 weeks | 7 | -1.414 | 0.157 | 0.314 |
| | | 2 weeks vs. 8 weeks | 7 | -1.000 | 0.317 | 0.317 |
| | A(H1N1) | <72 hours vs. 2 weeks | 8 | -2.207 | **0.027** | 0.054 |
| | | <72 hours vs. 8 weeks | 7 | -2.375 | **0.018** | 0.054 |
| | | 2 weeks vs. 8 weeks | 7 | -0.954 | 0.340 | 0.340 |

* Exact 2-tailed P-values determined by Wilcoxon signed rank test; ** Holm-Bonferroni adjusted P-values; P-values <0.05 are depicted in bold

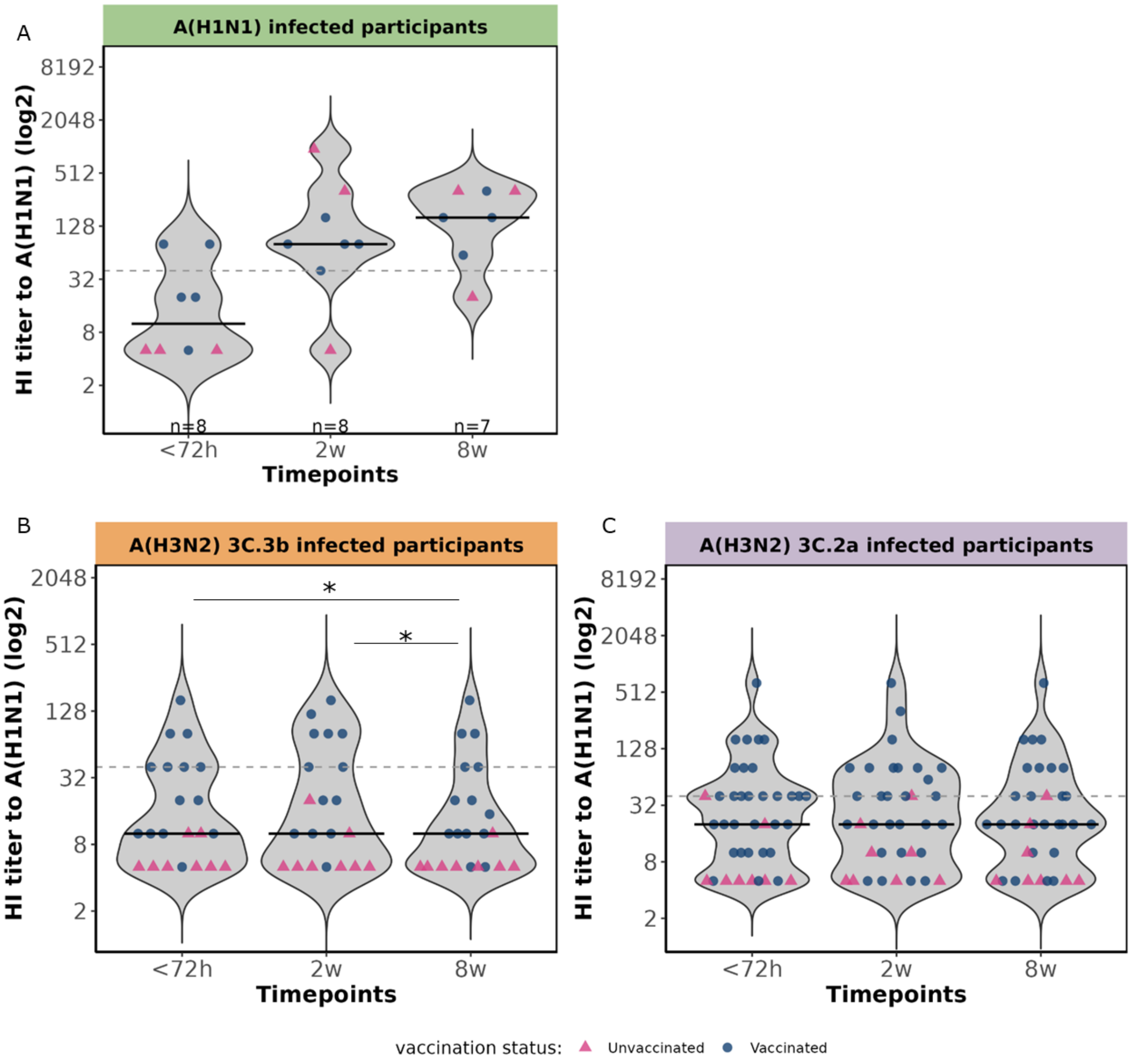


**Figure S2: The effect of infection strain on HI titers against A(H1N1).** In these figures the HI titers against the 2014/15 H1N1 strain are depicted in individuals with a (A) A(H1N1) infection and (B) a A(H3N2) 3C.3b or (C) A(H3N2) 3C.2a infection across three timepoints. Medians are indicated by the black line. The grey dashed line indicates an HI titer of 40. Unvaccinated individuals are indicated in pink triangles. P-values were determined by Wilcoxon signed rank test: *P < 0.05; **P < 0.01; ***P < 0.001; ****P < 0.0001.

**Table S6: Mann-Whitney U test comparing HI responses in vaccinated vs. unvaccinated individuals within the infection strain**

| Influenza virus infection strain | Titers against | Time point | group | N | Mean rank | U | Z | P-value* | Adjusted P-value** |
|---|---|---|---|---|---|---|---|---|---|
| **A(H3N2) 3C.3b** | A(H3N2) 3C.1 2014/15 vaccine strain | <72 hours | vaccinated | 14 | 13.6 | 27.0 | -2.118 | 0.050 | 0.150 |
| | | | unvaccinated | 8 | 7.9 | | | | |
| | | 2 weeks | vaccinated | 13 | 11.1 | 50.5 | -0.110 | 0.916 | 0.916 |
| | | | unvaccinated | 8 | 10.8 | | | | |
| | | 8 weeks | vaccinated | 14 | 12.4 | 43.0 | -0.897 | 0.402 | 0.804 |
| | | | unvaccinated | 8 | 9.9 | | | | |
| | A(H3N2) 3C.3a 2015/16 vaccine strain | <72 hours | vaccinated | 14 | 12.8 | 37.5 | -1.421 | 0.212 | 0.424 |
| | | | unvaccinated | 8 | 9.2 | | | | |
| | | 2 weeks | vaccinated | 13 | 11.6 | 44.0 | -0.597 | 0.595 | 0.595 |
| | | | unvaccinated | 8 | 10.0 | | | | |
| | | 8 weeks | vaccinated | 14 | 14.3 | 17.0 | -2.704 | **0.006** | **0.019** |
| | | | unvaccinated | 8 | 6.6 | | | | |
| | A(H1N1) | <72 hours | vaccinated | 14 | 14.9 | 9.0 | -3.305 | **<0.001** | **0.002** |
| | | | unvaccinated | 8 | 5.6 | | | | |
| | | 2 weeks | vaccinated | 13 | 14.2 | 10.5 | -3.081 | **0.001** | **0.001** |
| | | | unvaccinated | 8 | 5.8 | | | | |
| | | 8 weeks | vaccinated | 14 | 14.7 | 11.0 | -3.203 | **0.001** | **0.002** |
| | | | unvaccinated | 8 | 5.9 | | | | |
| **A(H3N2) 3C.2a** | A(H3N2) 3C.1 2014/15 vaccine strain | <72 hours | vaccinated | 31 | 22.3 | 52.5 | -2.540 | **0.011** | **0.032** |
| | | | unvaccinated | 8 | 11.1 | | | | |
| | | 2 weeks | vaccinated | 28 | 20.3 | 61 | -1.963 | 0.053 | 0.107 |
| | | | unvaccinated | 8 | 12.1 | | | | |
| | | 8 weeks | vaccinated | 27 | 19.8 | 60 | -1.913 | 0.061 | 0.061 |
| | | | unvaccinated | 8 | 12.0 | | | | |
| | A(H3N2) 3C.2a | <72 hours | vaccinated | 31 | 21.9 | 66.5 | -2.072 | **0.044** | 0.132 |
| | | | unvaccinated | 8 | 12.8 | | | | |
| | | 2 weeks | vaccinated | 28 | 19.6 | 82 | -1.156 | 0.267 | 0.267 |
| | | | unvaccinated | 8 | 14.8 | | | | |
| | | 8 weeks | vaccinated | 27 | 19.7 | 61.5 | -1.879 | 0.067 | 0.134 |
| | | | unvaccinated | 8 | 12.2 | | | | |
| | A(H3N2) 3C.3a 2015/16 vaccine strain | <72 hours | vaccinated | 31 | 20.6 | 105.5 | -0.673 | 0.527 | 1.000 |
| | | | unvaccinated | 8 | 17.7 | | | | |
| | | 2 weeks | vaccinated | 28 | 18.8 | 102.5 | -0.371 | 0.723 | 0.723 |
| | | | unvaccinated | 8 | 17.3 | | | | |
| | | 8 weeks | vaccinated | 27 | 18.5 | 94.5 | -0.556 | 0.603 | 1.000 |
| | | | unvaccinated | 8 | 16.3 | | | | |
| | H1N1 | <72 hours | vaccinated | 31 | 22.8 | 38.5 | -3.032 | **0.002** | **0.005** |
| | | | unvaccinated | 8 | 9.3 | | | | |
| | | 2 weeks | vaccinated | 28 | 20.9 | 44.5 | -2.607 | **0.008** | **0.008** |
| | | | unvaccinated | 8 | 10.1 | | | | |
| | | 8 weeks | vaccinated | 27 | 20.4 | 42.5 | -2.633 | **0.008** | **0.016** |
| | | | unvaccinated | 8 | 9.8 | | | | |

* Exact 2-tailed P-values determined by Mann-Whitney U test; ** Holm-Bonferroni adjusted P-values; P-values <0.05 are depicted in bold

**Table S7: Participant characteristics of T cell response subset**

| | N | Median age in years (IQR) | Female, n (%) |
|---|---|---|---|
| **A(H1N1)** | 8 | 66 (64.3-69.5) | 4 (50%) |
| **A(H3N2)** | 64 | 69.5 (66-73.8) | 26 (40.6%) |
| **- 3C.2a** | 37 | 69 (66-73) | 17 (45.9%) |
| **- 3C.3b** | 20 | 67 (64.5-73.8) | 7 (35%) |
| **- Not typable** | 7 | 71 (66-78) | 2 (28.6%) |
| **P-value** | | 0.258[a] | 0.713[b] |

[a]Kruskal-Wallis; [b] Pearson Chi-square

**Table S8: Participant vaccination status of T cell response subset**

| | N | Median age in years (IQR) | Female, n (%) |
|---|---|---|---|
| **Vaccinated** | 53 | 70 (66-74) | 22 (41.5%) |
| **Unvaccinated** | 19 | 66 (63-68) | 8 (42.1%) |
| **P-value** | | **0.004**[a] | 0.964[b] |

[a] Mann-Whitney U; [b] Chi-square; P-values <0.05 are depicted in bold

**Table S9: Wilcoxon signed rank test of the IFN-y responses against HA peptide pools by vaccination status comparing timepoints**

| Vaccination status | Stimulus | Time point Pairs tested | N pairs | Z | P-value* | Adjusted P-value ** |
|---|---|---|---|---|---|---|
| **unvaccinated** | A(H3N2) 3C.1 14/15 vaccine strain | <72 hours vs. 2 weeks | 15 | -1.364 | 0.173 | 0.346 |
| | | <72 hours vs. 8 weeks | 16 | -1.917 | 0.055 | 0.165 |
| | | 2 weeks vs. 8 weeks | 17 | -0.063 | 0.950 | 0.950 |
| | A(H3N2) 3C.2a | <72 hours vs. 2 weeks | 15 | -1.696 | 0.090 | 0.180 |
| | | <72 hours vs. 8 weeks | 16 | -1.863 | 0.062 | 0.186 |
| | | 2 weeks vs. 8 weeks | 17 | -0.070 | 0.944 | 0.944 |
| | A(H3N2) 3C.3b | <72 hours vs. 2 weeks | 15 | -1.336 | 0.182 | 0.546 |
| | | <72 hours vs. 8 weeks | 16 | -0.672 | 0.501 | 0.501 |
| | | 2 weeks vs. 8 weeks | 17 | -0.931 | 0.352 | 0.704 |
| | A(H1N1) | <72 hours vs. 2 weeks | 15 | -0.578 | 0.563 | 1.000 |
| | | <72 hours vs. 8 weeks | 16 | -0.561 | 0.575 | 1.000 |
| | | 2 weeks vs. 8 weeks | 17 | -0.133 | 0.894 | 0.894 |
| | M1 | <72 hours vs. | 15 | -1.351 | 0.177 | 0.531 |

| | | | | | | |
|---|---|---|---|---|---|---|
| | | 2 weeks | | | | |
| | | <72 hours vs. 8 weeks | 16 | -1.119 | 0.263 | 0.526 |
| | | 2 weeks vs. 8 weeks | 17 | -0.408 | 0.683 | 0.683 |
| **vaccinated** | A(H3N2) 3C.1 14/15 vaccine strain | <72 hours vs. 2 weeks | 39 | -2.813 | **0.005** | **0.015** |
| | | <72 hours vs. 8 weeks | 41 | -2.346 | **0.019** | **0.038** |
| | | 2 weeks vs. 8 weeks | 42 | -0.988 | 0.323 | 0.323 |
| | A(H3N2) 3C.2a | <72 hours vs. 2 weeks | 39 | -2.996 | **0.003** | **0.009** |
| | | <72 hours vs. 8 weeks | 41 | -1.226 | 0.220 | 0.220 |
| | | 2 weeks vs. 8 weeks | 42 | -1.836 | 0.066 | 0.132 |
| | A(H3N2) 3C.3b | <72 hours vs. 2 weeks | 39 | -2.443 | **0.015** | **0.045** |
| | | <72 hours vs. 8 weeks | 41 | -0.718 | 0.473 | 0.473 |
| | | 2 weeks vs. 8 weeks | 42 | -1.827 | 0.068 | 0.136 |
| | A(H1N1) | <72 hours vs. 2 weeks | 39 | -1.858 | 0.063 | 0.189 |
| | | <72 hours vs. 8 weeks | 41 | -1.853 | 0.064 | 0.128 |
| | | 2 weeks vs. 8 weeks | 42 | -0.056 | 0.955 | 0.955 |
| | M1 | <72 hours vs. 2 weeks | 39 | -2.365 | **0.018** | **0.036** |
| | | <72 hours vs. 8 weeks | 41 | -1.284 | 0.199 | 0.199 |
| | | 2 weeks vs. 8 weeks | 42 | -2.675 | **0.007** | **0.021** |

* Exact 2-tailed P-values determined by Wilcoxon signed rank test; ** Holm-Bonferroni adjusted P-values; P-values <0.05 are depicted in bold

**Table S10: Mann-Whitney U test of the IFN-y response to HA peptide pools of influenza virus strains comparing vaccinated vs. unvaccinated**

| stimulus | Time point | group | N | Mean rank | U | Z | P-value* | Adjusted P-value** |
|---|---|---|---|---|---|---|---|---|
| HA A(H3N2) 3C.1 14/15 vaccine strain | <72 hours | vaccinated | 44 | 29.18 | 294.0 | -0.996 | 0.319 | 0.957 |
| | | unvaccinated | 16 | 34.13 | | | | |
| | 2 weeks | vaccinated | 48 | 34.89 | 365.5 | -0.963 | 0.336 | 0.336 |
| | | unvaccinated | 18 | 29.81 | | | | |
| | 8 weeks | vaccinated | 47 | 34.43 | 356.0 | -0.987 | 0.324 | 0.648 |
| | | unvaccinated | 18 | 29.28 | | | | |
| HA A(H1N1) | <72 hours | vaccinated | 44 | 30.93 | 333.0 | -0.363 | 0.717 | 0.717 |
| | | unvaccinated | 16 | 29.31 | | | | |
| | 2 weeks | vaccinated | 48 | 35.28 | 346.5 | -1.271 | 0.204 | 0.612 |
| | | unvaccinated | 18 | 28.75 | | | | |
| | 8 weeks | vaccinated | 47 | 34.19 | 367.0 | -0.865 | 0.387 | 0.774 |
| | | unvaccinated | 18 | 29.89 | | | | |
| HA A(H3N2) 3C.3b | <72 hours | vaccinated | 44 | 29.81 | 321.5 | -0.511 | 0.610 | 0.610 |
| | | unvaccinated | 16 | 32.41 | | | | |
| | 2 weeks | vaccinated | 48 | 34.75 | 372.0 | -0.864 | 0.387 | 1.000 |
| | | unvaccinated | 18 | 30.17 | | | | |
| | 8 weeks | vaccinated | 47 | 34.11 | 371.0 | -0.764 | 0.445 | 0.890 |
| | | unvaccinated | 18 | 30.11 | | | | |
| HA A(H3N2) 3C.2a | <72 hours | vaccinated | 44 | 30.49 | 351.5 | -0.009 | 0.993 | 0.993 |
| | | unvaccinated | 16 | 30.53 | | | | |
| | 2 weeks | vaccinated | 48 | 34.81 | 369.0 | -0.914 | 0.361 | 1.000 |
| | | unvaccinated | 18 | 30.00 | | | | |
| | 8 weeks | vaccinated | 47 | 32.50 | 399.5 | -0.349 | 0.727 | 1.000 |
| | | unvaccinated | 18 | 34.31 | | | | |

* P-values determined by Mann-Whitney U test; ** Holm-Bonferroni adjusted P-values

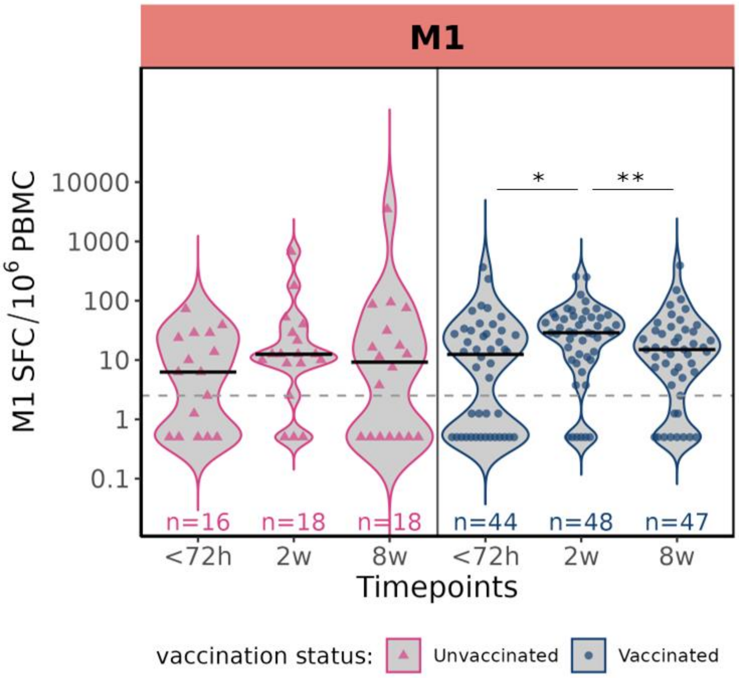


**Figure S3: The effect of infection on longitudinal influenza virus-specific T cell responses to M1 stratified by vaccination status.** In this figure the T cell response as determined by IFN-y ELISpot is depicted. The IFN-y response after PBMC stimulation with an influenza virus peptide pool of M1 is depicted in individuals with an influenza virus infection comparing vaccinated versus unvaccinated individuals. Total samples used per time point were, n = 60 , n = 66 and n = 65 for timepoints <72 hours, 2 weeks or 8 weeks after infection respectively. Medians are indicated by the black line. The dashed line indicates a response of 2.5 SFC/$10^6$ PBMC. P-values were determined by Wilcoxon signed rank test: *P < 0.05; **P < 0.01; ***P < 0.001; ****P < 0.0001.

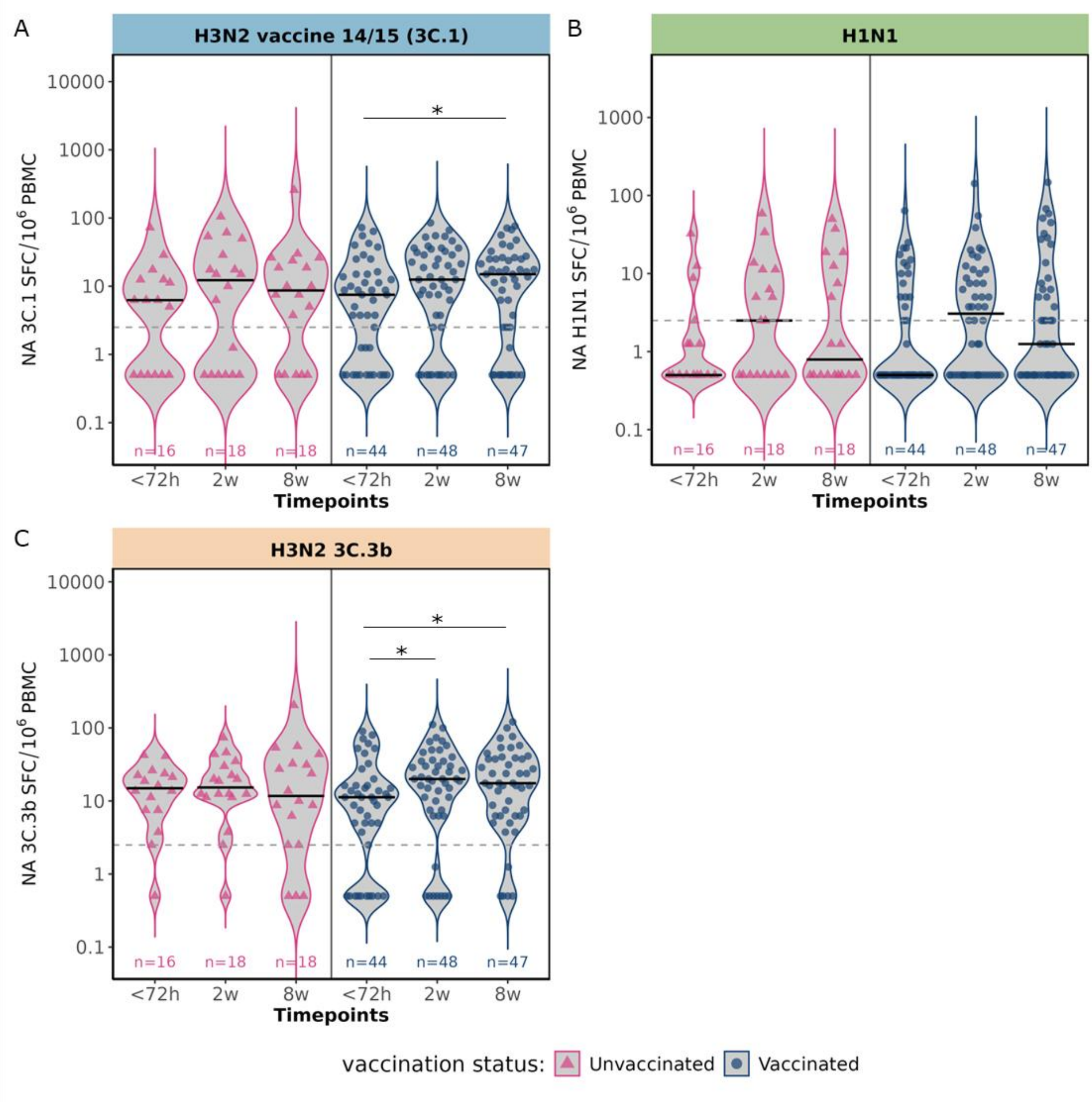


**Figure S4: The effect of infection on longitudinal influenza virus-specific T cell responses against NA stratified by vaccination status.** In this figure the T cell response as determined by IFN-γ ELISpot is depicted. The IFN-γ response after PBMC stimulation with influenza virus peptide pools of Neuraminidase (NA) of (A) the A(H3N2) 3C.1 and (B) A(H1N1) 2014/15 vaccine strains and the circulating (C) A(H3N2) strain 3C.3b is depicted, in individuals with an influenza virus infection, comparing vaccinated versus unvaccinated individuals. Total samples used per time point were, n = 60 , n = 66 and n = 65 for timepoints <72 hours, 2 weeks or 8 weeks after infection respectively. Medians are indicated by the black line. The dashed line indicates a response of 2.5 SFC/$10^6$ PBMC. P-values as determined by Wilcoxon signed rank test: *P < 0.05; **P < 0.01; ***P < 0.001; ****P < 0.0001.

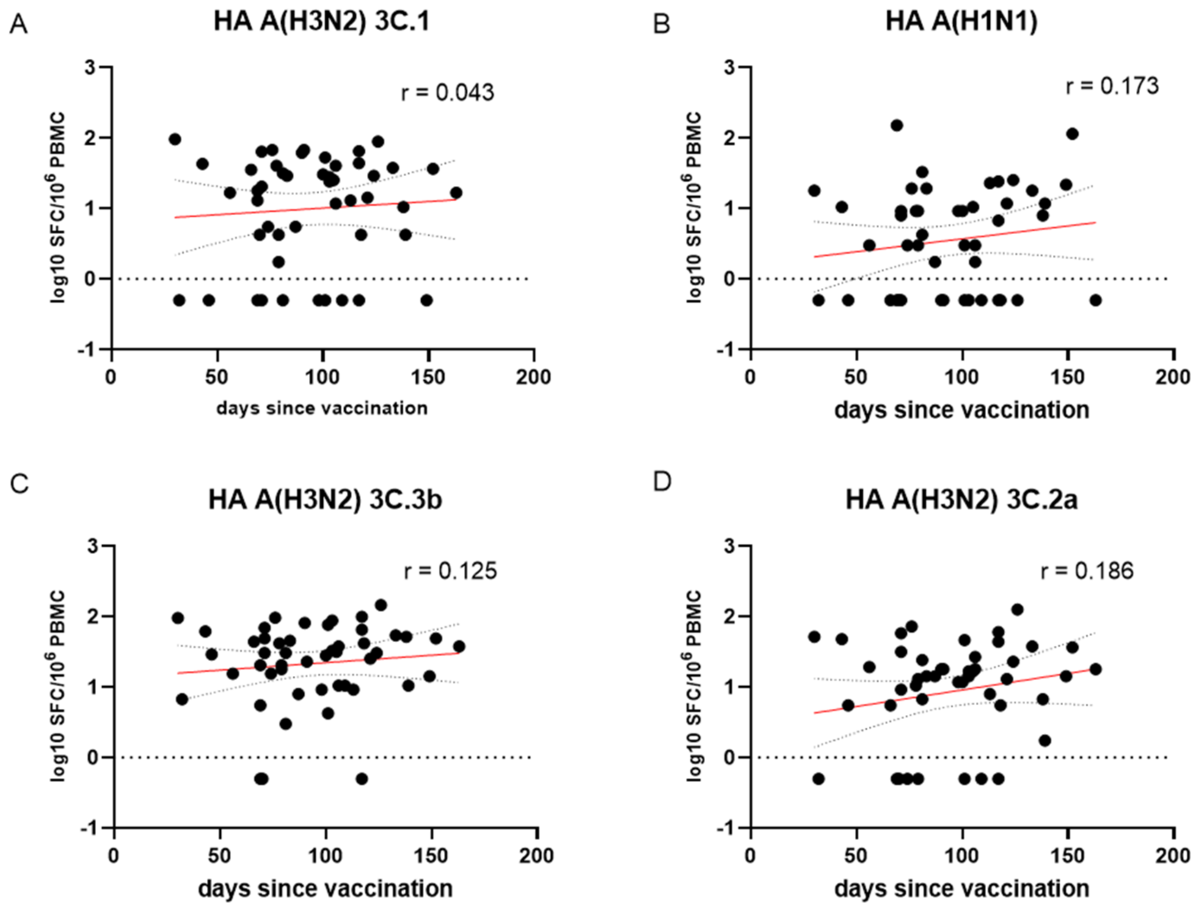


**Figure S5: Correlation between IFN-y levels and the number of days since vaccination.** In this figure the correlations between the days since vaccination and the IFN-y levels against the (A) 2014/15 A(H3N2) 3C.1 vaccine strain, (B) A(H1N1) strain, (C) A(H3N2) 3C.3b vaccine strain and the circulating A(H3N2) 3C.2a strain are depicted. Spearman r-coefficients are depicted in the plots

**Table S11: Wilcoxon signed rank test of the IFN-y responses against HA peptide pools comparing timepoints within the infection strain**

| Infection strain | stimulus | Time point Pairs tested | N | Z | P-value* | Adjusted P-value ** |
|---|---|---|---|---|---|---|
| **A(H3N2) 3C.3b** | HA A(H3N2) 3C.1 14/15 vaccine strain | <72 hours vs. 2 weeks | 12 | -0.237 | 0.812 | 0.812 |
| | | <72 hours vs. 8 weeks | 18 | -1.024 | 0.306 | 0.918 |
| | | 2 weeks vs. 8 weeks | 12 | -0.450 | 0.653 | 1.000 |
| | HA A(H3N2) 3C.2a | <72 hours vs. 2 weeks | 12 | -0.471 | 0.638 | 1.000 |
| | | <72 hours vs. 8 weeks | 18 | -0.153 | 0.878 | 1.000 |
| | | 2 weeks vs. 8 weeks | 12 | -0.142 | 0.887 | 0.887 |
| | HA A(H3N2) 3C.3b | <72 hours vs. 2 weeks | 12 | -0.628 | 0.530 | 1.000 |
| | | <72 hours vs. 8 weeks | 18 | -0.311 | 0.756 | 0.756 |
| | | 2 weeks vs. 8 weeks | 12 | -0.959 | 0.338 | 1.000 |
| | HA A(H1N1) | <72 hours vs. 2 weeks | 12 | -1.246 | 0.213 | 0.639 |
| | | <72 hours vs. 8 weeks | 18 | -0.612 | 0.540 | 1.000 |
| | | 2 weeks vs. 8 weeks | 12 | -0.118 | 0.906 | 0.906 |
| **A(H3N2) 3C.2a** | HA A(H3N2) 3C.1 14/15 vaccine strain | <72 hours vs. 2 weeks | 30 | -3.071 | **0.002** | **0.006** |
| | | <72 hours vs. 8 weeks | 31 | -2.012 | **0.044** | 0.088 |
| | | 2 weeks vs. 8 weeks | 29 | -0.968 | 0.333 | 0.333 |
| | HA A(H3N2) 3C.2a | <72 hours vs. 2 weeks | 30 | -3.394 | **<0.001** | **0.002** |
| | | <72 hours vs. 8 weeks | 31 | -1.615 | 0.106 | 0.212 |
| | | 2 weeks vs. 8 weeks | 29 | -1.512 | 0.131 | 0.131 |
| | HA A(H3N2) 3C.3b | <72 hours vs. 2 weeks | 30 | -1.903 | 0.057 | 0.171 |
| | | <72 hours vs. 8 weeks | 31 | -0.010 | 0.992 | 0.992 |
| | | 2 weeks vs. 8 weeks | 29 | -1.778 | 0.075 | 0.151 |
| | HA A(H1N1) | <72 hours vs. 2 weeks | 30 | -1.591 | 0.112 | 0.335 |
| | | <72 hours vs. 8 weeks | 31 | -1.427 | 0.154 | 0.308 |
| | | 2 weeks vs. 8 weeks | 29 | -0.374 | 0.709 | 0.709 |
| **A(H1N1)** | HA A(H3N2) 3C.1 14/15 vaccine strain | <72 hours vs. 2 weeks | 6 | -0.677 | 0.498 | 1.000 |
| | | <72 hours vs. 8 weeks | 7 | -0.949 | 0.343 | 1.000 |

| | | | | | |
|---|---|---|---|---|---|
| | 2 weeks vs. 8 weeks | 6 | 0.000 | 1.000 | 1.000 |
| HA A(H3N2) 3C.2a | <72 hours vs. 2 weeks | 6 | -1.214 | 0.225 | 0.675 |
| | <72 hours vs. 8 weeks | 7 | -0.946 | 0.344 | 0.688 |
| | 2 weeks vs. 8 weeks | 6 | -0.734 | 0.463 | 0.463 |
| HA A(H3N2) 3C.3b | <72 hours vs. 2 weeks | 6 | -0.946 | 0.344 | 0.688 |
| | <72 hours vs. 8 weeks | 7 | -1.153 | 0.249 | 0.747 |
| | 2 weeks vs. 8 weeks | 6 | -0.105 | 0.917 | 0.917 |
| HA A(H1N1) | <72 hours vs. 2 weeks | 6 | -2.023 | **0.043** | 0.129 |
| | <72 hours vs. 8 weeks | 7 | -1.826 | 0.068 | 0.136 |
| | 2 weeks vs. 8 weeks | 6 | -1.051 | 0.293 | 0.293 |

* Exact 2-tailed P-values determined by Wilcoxon signed rank test; ** Holm-Bonferroni adjusted P-values; P-values <0.05 are depicted in bold

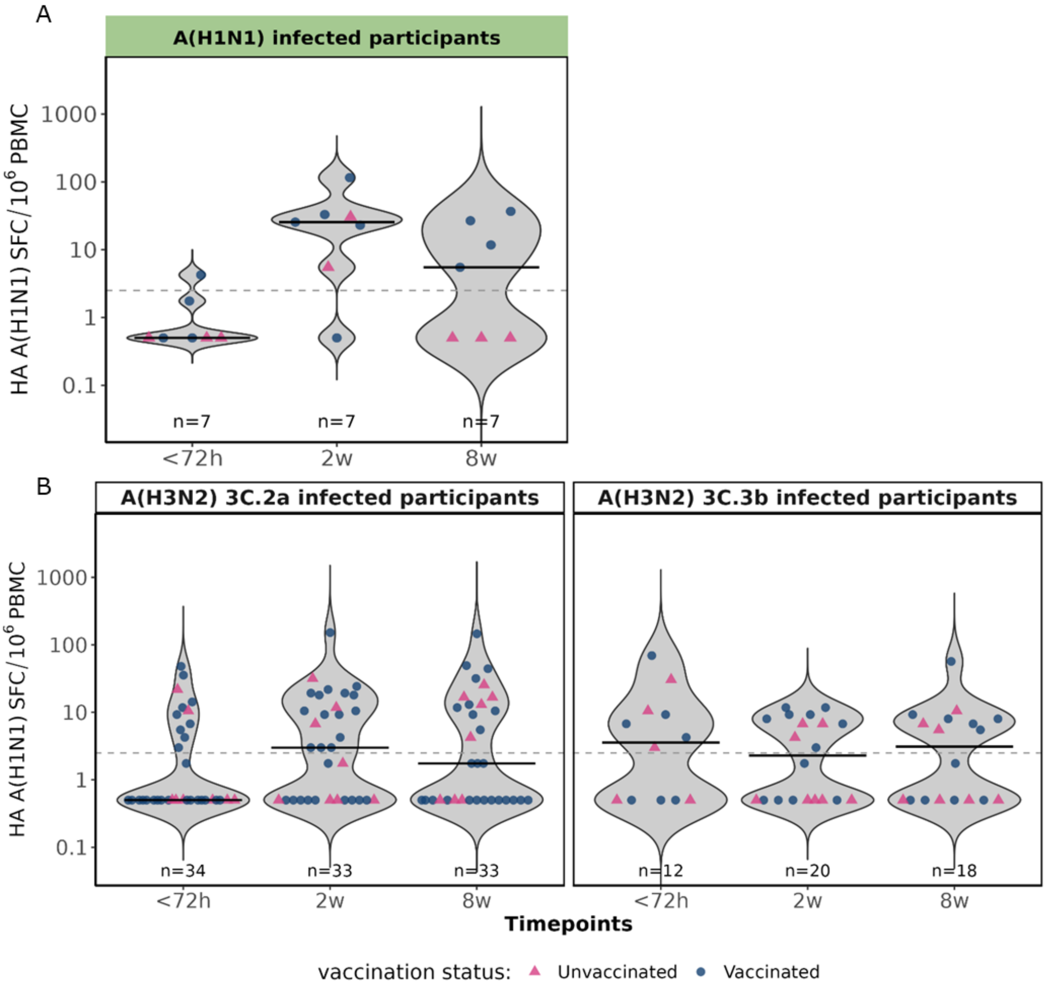


**Figure S6: The effect of infection strain on influenza virus-specific T cell responses against H1N1.** In these figures the IFN-γ T cell response in PBMC stimulated with Hemagglutinin (HA) of the 2014/15 A(H1N1) strain are depicted for individuals with an (A) A(H1N1) infection and (B) an A(H3N2) 3C.3b or A(H3N2) 3C.2a infection, across three timepoints. Medians are indicated by the black line. The dashed line indicates a response of 2.5 SFC/$10^6$ PBMC.

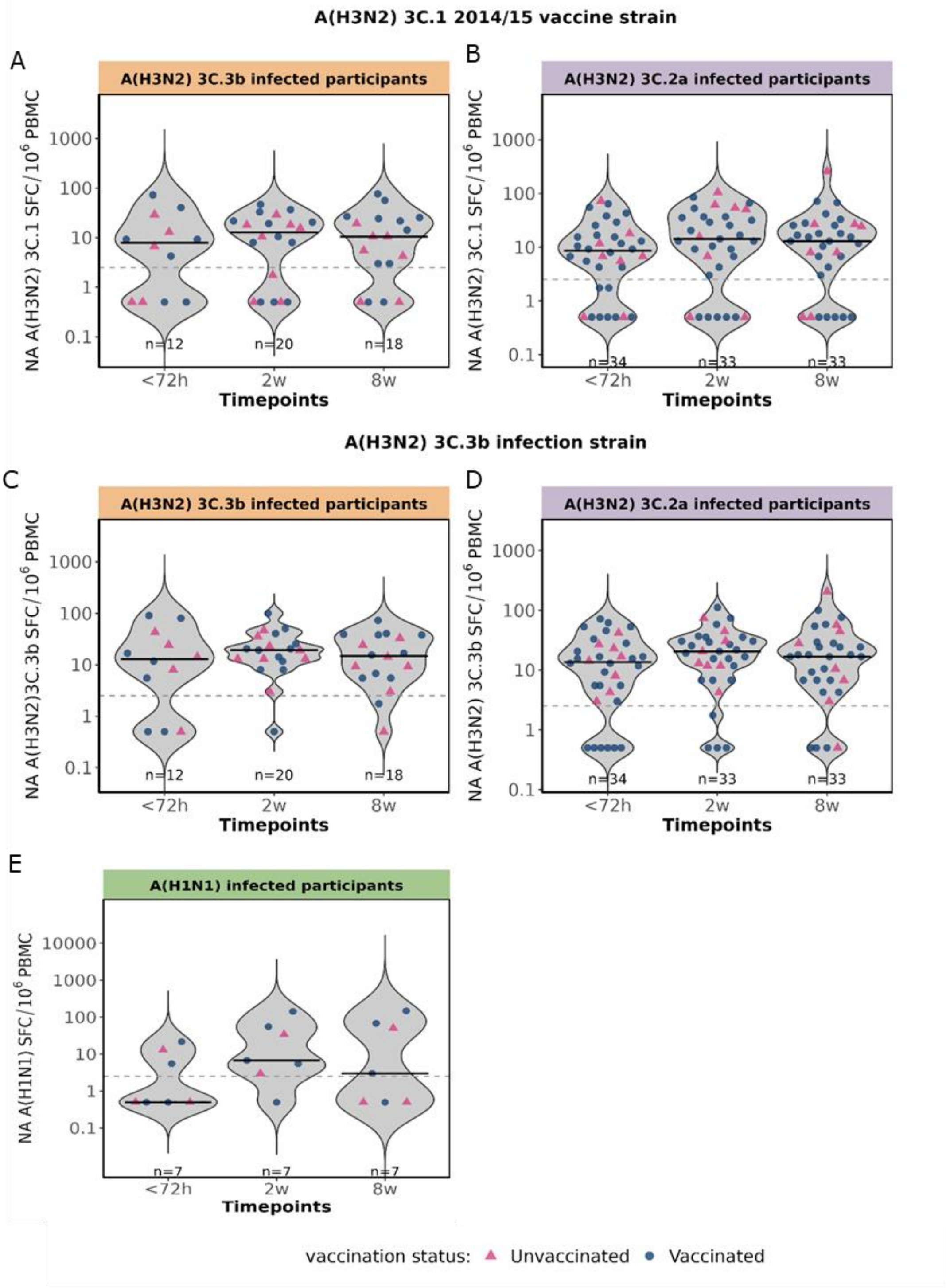


**Figure S7: influenza virus-specific T cell responses to neuraminidase peptide pools of vaccine and infection strains in older adults with influenza virus infection.** In this figure the (A-B) T cell responses against neuraminidase (NA) of the A(H3N2) vaccine strain and (C-D) A(H3N2) circulating strain 3C.3b are depicted in individuals infected with A(H3N2) 3C.3b and A(H3N2) 3C.2a, as well as (E) the response to A(H1N1) in individuals infected with A(H1N1). Unvaccinated individuals are shown in pink. The dashed line indicates a response of 2.5 SFC/$10^6$ PBMC. Medians are indicated by the black line.

**Table S12: Mann-Whitney U test of the IFN-y responses against HA peptide pools comparing vaccinated vs. unvaccinated individuals within the infection strain**

| Influenza virus infection strain | Stimulus | Time point | group | N | Mean rank | U | Z | P-value* | Adjusted P-value ** |
|---|---|---|---|---|---|---|---|---|---|
| **A(H3N2) 3C.3b** | HA A(H3N2) 3C.1 | <72 hours | vaccinated | 7 | 6.4 | 17.0 | -0.083 | 1.000 | 1.000 |
| | | | unvaccinated | 5 | 6.6 | | | | |
| | | 2 weeks | vaccinated | 12 | 10.3 | 46.0 | -0.156 | 0.910 | 1.000 |
| | | | unvaccinated | 8 | 10.8 | | | | |
| | | 8 weeks | vaccinated | 11 | 11.3 | 19.0 | -1.776 | 0.085 | 0.255 |
| | | | unvaccinated | 7 | 6.7 | | | | |
| | HA A(H3N2) 3C.2a | <72 hours | vaccinated | 7 | 6.6 | 17.0 | -0.082 | 1.000 | 1.000 |
| | | | unvaccinated | 5 | 6.4 | | | | |
| | | 2 weeks | vaccinated | 12 | 10.8 | 44.0 | -0.311 | 0.792 | 1.000 |
| | | | unvaccinated | 8 | 10.0 | | | | |
| | | 8 weeks | vaccinated | 11 | 10.5 | 27.0 | -1.062 | 0.328 | 0.984 |
| | | | unvaccinated | 7 | 7.9 | | | | |
| | HA A(H3N2) 3C.3b | <72 hours | vaccinated | 7 | 6.0 | 14.0 | -0.570 | 0.639 | 0.639 |
| | | | unvaccinated | 5 | 7.2 | | | | |
| | | 2 weeks | vaccinated | 12 | 11.3 | 38.5 | -0.734 | 0.473 | 0.946 |
| | | | unvaccinated | 8 | 9.3 | | | | |
| | | 8 weeks | vaccinated | 11 | 10.6 | 26.5 | -1.090 | 0.285 | 0.855 |
| | | | unvaccinated | 7 | 7.8 | | | | |
| | HA A(H1N1) | <72 hours | vaccinated | 7 | 6.4 | 17.0 | -0.084 | 1.000 | 1.000 |
| | | | unvaccinated | 5 | 6.6 | | | | |
| | | 2 weeks | vaccinated | 12 | 12.1 | 29.0 | -1.541 | 0.157 | 0.471 |
| | | | unvaccinated | 8 | 8.1 | | | | |
| | | 8 weeks | vaccinated | 11 | 10.3 | 30.0 | -0.807 | 0.479 | 0.958 |
| | | | unvaccinated | 7 | 8.3 | | | | |
| **A(H3N2) 3C.2a** | HA A(H3N2) 3C.1 | <72 hours | vaccinated | 26 | 16.5 | 77.5 | -1.101 | 0.288 | 0.864 |
| | | | unvaccinated | 8 | 20.8 | | | | |
| | | 2 weeks | vaccinated | 25 | 17.0 | 99.5 | -0.021 | 0.984 | 0.984 |
| | | | unvaccinated | 8 | 17.1 | | | | |
| | | 8 weeks | vaccinated | 25 | 16.3 | 81.5 | -0.780 | 0.445 | 0.889 |
| | | | unvaccinated | 8 | 19.3 | | | | |
| | HA A(H3N2) 3C.2a | <72 hours | vaccinated | 26 | 17.4 | 102.0 | -0.083 | 0.952 | 0.952 |
| | | | unvaccinated | 8 | 17.8 | | | | |
| | | 2 weeks | vaccinated | 25 | 16.9 | 97.5 | -0.106 | 0.918 | 1.000 |
| | | | unvaccinated | 8 | 17.3 | | | | |
| | | 8 weeks | vaccinated | 25 | 16.0 | 74.0 | -1.113 | 0.290 | 0.870 |
| | | | unvaccinated | 8 | 20.3 | | | | |
| | HA A(H3N2) 3C.3b | <72 hours | vaccinated | 26 | 17.8 | 96.0 | -0.325 | 0.765 | 1.000 |
| | | | unvaccinated | 8 | 16.5 | | | | |
| | | 2 weeks | vaccinated | 25 | 17.0 | 99.5 | -0.021 | 0.984 | 0.984 |
| | | | unvaccinated | 8 | 17.1 | | | | |
| | | 8 weeks | vaccinated | 25 | 17.3 | 92.0 | -0.337 | 0.757 | 1.000 |
| | | | unvaccinated | 8 | 16.0 | | | | |
| | HA A(H1N1) | <72 hours | vaccinated | 26 | 17.9 | 94.0 | -0.475 | 0.705 | 0.705 |
| | | | unvaccinated | 8 | 16.3 | | | | |
| | | 2 weeks | vaccinated | 25 | 17.7 | 83.5 | -0.716 | 0.496 | 0.992 |
| | | | unvaccinated | 8 | 14.9 | | | | |
| | | 8 weeks | vaccinated | 25 | 16.2 | 81.0 | -0.848 | 0.445 | 1.000 |
| | | | unvaccinated | 8 | 19.4 | | | | |

* Exact 2-tailed P-values determined by Mann-Whitney U test; ** Holm-Bonferroni adjusted P-values; P-values <0.05 are depicted in bold

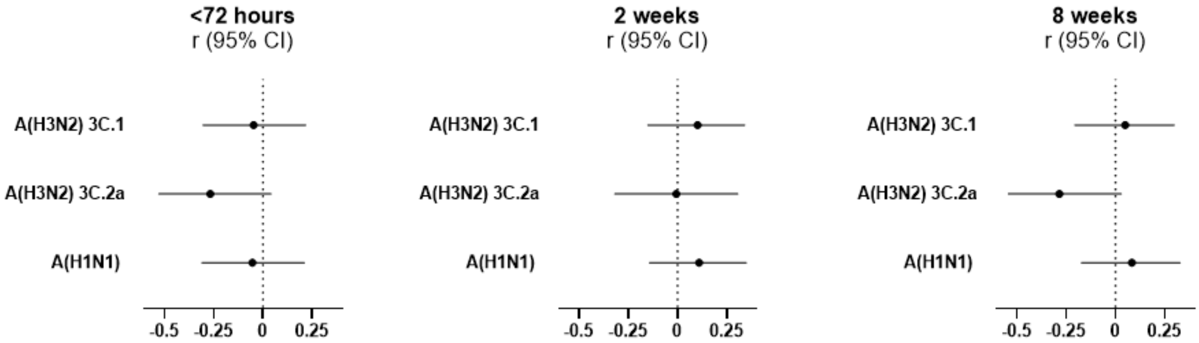


**Figure S8: Correlations between influenza virus-specific HI titers and IFN-y levels.** In this plot the Spearman r - coefficients and 95% confidence intervals are depicted for the correlations between HI titers and IFN-y levels per strain per timepoint.

**Table S13: Spearman correlations between symptoms and HI titers against influenza A virus strains at 2 weeks post-infection**

| | Influenza virus strain | N pairs | Spearman r (95% CI) | P-value | Adjusted P-value* |
|---|---|---|---|---|---|
| **Number of Symptoms** | A(H3N2) 3C.1 2014/15 vaccine strain | 93 | -0.0196 (-0.228 to 0.191) | 0.852 | 0.852 |
| | A(H3N2) 3C.3a 2015/16 vaccine strain | 93 | -0.028 (-0.236 to 0.182) | 0.789 | 1.000 |
| | A(H3N2) 3C.2a | 46 | 0.298 (-0.001 to 0.547) | **0.045** | 0.180 |
| | A(H1N1) | 93 | 0.071 (-0.140 to 0.277) | 0.498 | 1.000 |
| **Symptom duration**** | A(H3N2) 3C.1 2014/15 vaccine strain | 92 | 0.080 (-0.133 to 0.286) | 0.449 | 0.898 |
| | A(H3N2) 3C.3a 2015/16 vaccine strain | 92 | 0.057 (-0.156 to 0.264) | 0.593 | 0.593 |
| | A(H3N2) 3C.2a | 45 | 0.227 (-0.081 to 0.495) | 0.134 | 0.536 |
| | A(H1N1) | 92 | 0.147 (-0.066 to 0.347) | 0.162 | 0.486 |
| **Fever duration**** | A(H3N2) 3C.1 2014/15 vaccine strain | 92 | -0.022 (-0.231 to 0.190) | 0.836 | 1.000 |
| | A(H3N2) 3C.3a 2015/16 vaccine strain | 92 | 0.019 (-0.192 to 0.229) | 0.855 | 0.855 |
| | A(H3N2) 3C.2a | 45 | 0.108 (-0.200 to 0.397) | 0.480 | 1.000 |
| | A(H1N1) | 92 | -0.030 (-0.239 to 0.182) | 0.774 | 1.000 |

* Holm-Bonferroni adjusted P-values; significant P-values depicted in bold; **Duration was measured in days

**Table S14: Spearman correlations between symptoms and IFN-γ levels against HA of influenza A virus strains at 2 weeks post-infection**

| | Influenza virus strain | N pairs | Spearman r (95% CI) | P-value | Adjusted P-value* |
|---|---|---|---|---|---|
| **Number of Symptoms** | A(H3N2) 3C.1 2014/15 vaccine strain | 66 | 0.191 (-0.061–0.420) | 0.125 | 0.250 |
| | A(H3N2) 3C.3b | 66 | 0.144 (-0.109 – 0.379) | 0.249 | 0.249 |
| | A(H3N2) 3C.2a | 66 | 0.281 (0.034 – 0.495) | **0.022** | 0.088 |
| | A(H1N1) | 66 | 0.245 (-0.004 – 0.466) | **0.047** | 0.141 |
| **Symptom duration**** | A(H3N2) 3C.1 2014/15 vaccine strain | 65 | 0.034 (-0.219 – 0.282) | 0.791 | 0.791 |
| | A(H3N2) 3C.3b | 65 | 0.109 (-0.146 – 0.350) | 0.387 | 1.000 |
| | A(H3N2) 3C.2a | 65 | 0.097 (-0.158 – 0.339) | 0.443 | 1.000 |
| | A(H1N1) | 65 | 0.048 (-0.205 – 0.296) | 0.702 | 1.000 |
| **Fever duration**** | A(H3N2) 3C.1 2014/15 vaccine strain | 65 | -0.085 (-0.329 – 0.169) | 0.501 | 0.501 |
| | A(H3N2) 3C.3b | 65 | -0.094 (-0.337 – 0.160) | 0.454 | 0.908 |
| | A(H3N2) 3C.2a | 65 | -0.116 (-0.357 – 0.139) | 0.357 | 1.000 |
| | A(H1N1) | 65 | -0.255 (-0.476 – -0.005) | **0.040** | 0.160 |

* Holm-Bonferroni adjusted P-values; significant P-values depicted in bold; **Duration was measured in days

**Table S15: Mann-Whitney U test of the Influenza virus infection burden comparing vaccinated vs. unvaccinated**

| **Symptoms** | **group** | **N** | **Mean rank** | **U** | **Z** | **P-value*** | **Adjusted P-value **** |
|---|---|---|---|---|---|---|---|
| **Number of symptoms** | vaccinated | 69 | 49.71 | 1015.0 | -0.419 | 0.675 | 0.675 |
| | unvaccinated | 31 | 52.26 | | | | |
| **Symptom duration** | vaccinated | 67 | 48.13 | 947.0 | -0.700 | 0.484 | 0.968 |
| | unvaccinated | 31 | 52.45 | | | | |
| **Fever duration** | vaccinated | 67 | 45.75 | 787.0 | -1.939 | 0.052 | 0.156 |
| | unvaccinated | 31 | 57.61 | | | | |

* P-values determined by Mann-Whitney U test; ** Holm-Bonferroni adjusted P-values; duration was measured in days
